# FABP7 Regulates Acetyl-CoA Metabolism Through the Interaction with ACLY in the Nucleus of Astrocytes

**DOI:** 10.1007/s12035-020-02057-3

**Published:** 2020-08-19

**Authors:** Yoshiteru Kagawa, Banlanjo Abdulaziz Umaru, Hiroki Shima, Ryo Ito, Ryo Zama, Ariful Islam, Shin-ichiro Kanno, Akira Yasui, Shun Sato, Kosuke Jozaki, Subrata Kumar Shil, Hirofumi Miyazaki, Shuhei Kobayashi, Yui Yamamoto, Hiroshi Kogo, Chie Shimamoto-Mitsuyama, Akira Sugawara, Norihiro Sugino, Masayuki Kanamori, Teiji Tominaga, Takeo Yoshikawa, Kohji Fukunaga, Kazuhiko Igarashi, Yuji Owada

**Affiliations:** 1 Department of Organ Anatomy, Tohoku University Graduate School of Medicine, Sendai, 980-8575 Japan; 2 Department of Biochemistry, Tohoku University Graduate School of Medicine, Sendai, 980-8575 Japan; 3 Department of Molecular Endocrinology, Tohoku University Graduate School of Medicine, Sendai, 980-8575 Japan; 4 Division of Dynamic Proteome in Aging and Cancer, Institute of Development, Aging and Cancer (IDAC), Tohoku University, Sendai, 980-8575 Japan; 5 Department of Obstetrics and Gynecology, Yamaguchi University Graduate School of Medicine, Ube, 755-0046 Japan; 6 Department of Anatomy and Cell Biology, Gunma University Graduate School of Medicine, Maebashi, 371-8511 Japan; 7 Laboratory for Molecular Psychiatry, RIKEN Center for Brain Science, Wako, 351-0198 Japan; 8 Department of Neurosurgery, Tohoku University Graduate School of Medicine, Sendai, 980-8575 Japan; 9 Department of Pharmacology, Tohoku University Graduate School of Pharmaceutical Sciences, Sendai, 980-8578 Japan

**Keywords:** Fatty acid–binding protein (FABP), ATP-citrate lyase (ACLY), Acetyl-CoA, Histone acetylation, Caveolin-1, Astrocyte

## Abstract

Fatty acid binding protein 7 (FABP7) is an intracellular fatty acid chaperon that is highly expressed in astrocytes, oligodendrocyte-precursor cells, and malignant glioma. Previously, we reported that FABP7 regulates the response to extracellular stimuli by controlling the expression of caveolin-1, an important component of lipid raft. Here, we explored the detailed mechanisms underlying FABP7 regulation of caveolin-1 expression using primary cultured FABP7-KO astrocytes as a model of loss of function and NIH-3T3 cells as a model of gain of function. We discovered that FABP7 interacts with ATP-citrate lyase (ACLY) and is important for acetyl-CoA metabolism in the nucleus. This interaction leads to epigenetic regulation of several genes, including caveolin-1. Our novel findings suggest that FABP7-ACLY modulation of nuclear acetyl-CoA has more influence on histone acetylation than cytoplasmic acetyl-CoA. The changes to histone structure may modify caveolae-related cell activity in astrocytes and tumors, including malignant glioma.

**Electronic supplementary material:**

The online version of this article (10.1007/s12035-020-02057-3) contains supplementary material, which is available to authorized users.

## Introduction

Acetyl-coenzyme A (Acetyl-CoA) is an important metabolite that plays key roles in lipid biosynthesis, cell signaling, and epigenetics [1–3]. Synthesis of acetyl-CoA in the mitochondria occurs through oxidative decarboxylation of pyruvate in the TCA cycle and from β-oxidation of fatty acids. The metabolite is also synthesized from amino acids in the cytoplasm and nucleus. Synthesized acetyl-CoA is utilized for the generation of de novo fatty acids by several enzymes such as acetyl-CoA carboxylase (ACC) and fatty acid synthase (FASN), as well as for the essential acetyl donor including lysine acetyltransferases (KATs) [2], and it has been reported that the levels of newly generated acetyl-CoA in the nucleus are correlated with levels of histone acetylation [4].

Nuclear acetyl-CoA is mainly generated from the following: (1) glucose-oxidation-derived mitochondrial citrate through the action of ATP-citrate lyase (ACLY), (2) acetate through the action of acyl-coenzyme A synthetase short-chain family member 2 (ACSS2), (3) nuclear pyruvate through the action of pyruvate dehydrogenase complex (PDC) [5, 6]. All three enzymes are present in both the nucleus and cytosol and function in nucleus when they are required. It is reported that nuclear ACLY, but not cytoplasmic ACLY, is dynamically phosphorylated at S455 upon exposure to ionizing radiation and promotes homologous recombination through the KAT pathway [3] and that loss of nuclear ACSS2 in mouse hippocampus suppressed expression of memory-related genes through effects on histone H3 and H4 acetylation, thus impairing spatial memory formation [7]. Furthermore, it has been reported that, in eukaryotes, the biosynthesis of acetyl-CoA is thought to occur in the subcellular compartment where it is required, because it is membrane impermeable and very unstable due to the high-energy thioester bond that joins the acetyl and CoA groups [6]. Thus, acetyl-CoA may have spatiotemporal roles in cytoplasm and nucleus, respectively.

It is well known that diet and nutrition can alter the epigenetic state of the genome and affect gene expression by modifying DNA methylation and histone acetylation patterns [8]. Multiple studies have suggested that these alterations heighten risk of diseases including cancer, metabolic diseases, cardiovascular disease, developmental disorders, and mood disorders [9, 10]. For example, overfeeding of neonatal rats altered DNA methylation levels on the hypothalamic proopiomelanocortin gene, resulting in metabolic syndrome (obesity, hyperleptinemia, hyperinsulinemia, insulin resistance, and diabetes) [11]. In adult mice, a high-fat diet altered levels of DNA methylation and histone acetylation/methylation on dopaminergic and opioid genes [12]. On the other hand, polyunsaturated fatty acids (PUFAs) included in diet are shown to be some of the most important epigenetic regulators. Maternal intake of α-linolenic acid (ALA) influences postnatal development via regulating Fads DNA methylation in both maternal and offspring livers [13]. Furthermore, eicosapentaenoic acid (EPA) suppresses cell proliferation through demethylating tumor suppressor CCAAT/enhancer-binding proteins in U937 leukemia cells [14]. Despite these findings, we still know very little about the specific biological mechanisms behind PUFA alteration of epigenetic status.

Because PUFAs are insoluble in water, they require a carrier to function within cells. Fatty acid binding proteins (FABPs), found in both the nucleus and cytoplasm, solubilize PUFAs and control their uptake, metabolism, and intracellular storage [15]. It has been postulated that nuclear FABPs are involved in regulating transcriptional activity because they deliver PUFAs to nuclear receptors that act as transcription factors, such as peroxisome proliferator–activated receptors (PPARs) [16, 17]. However, little data are available on the relationship between epigenetic changes and the FABP-controlled dynamics of intracellular PUFAs.

In the brain, FABP7 is expressed by neural stem cells, astrocytes, and oligodendrocyte precursors [18–20], while in the liver, they are expressed by Kupffer cells [21]. FABP7 has high affinity for n-3 PUFAs, such as docosahexaenoic acid (DHA) and EPA [22]. In terms of function, FABP7 is involved in astrocyte proliferation [19] and malignant glioma migration [16]. In the process of elucidating its mechanism, we found that FABP7 in astrocytes controls the function of caveolae, a type of lipid raft and main source of cellular activity in response to external stimuli, via transcriptionally regulating the expression of caveolin-1, which is a key molecule for caveolae formation [23]. Caveolin-1 expression is regulated by transcription factors such as Evi-1, GATA6, ETS, HNF3/Fkh, AP4, AP2, SP1, and FOXO [24–27]. Additionally, DNA methylation and histone modifications in the caveolin-1 promoter are crucial for caveolin-1 transcription. Differences in epigenetic state on caveolin-1 promoter could lead to differentiation of adipocyte [28] and colon cancer [29], as well as migration and invasion of breast cancer [30]. Taken together, these results suggest that FABP7-regulated lipid raft function may influence cellular activity via epigenetic regulation of caveolin-1.

Here, we explored detailed mechanisms underlying FABP7 regulation of caveolin-1. We used primary cultured FABP7-KO astrocytes as a loss-of-function model, and NIH-3T3 cells as a gain-of-function model. We showed that FABP7 interacts with ACLY. This interaction regulates nuclear acetyl-CoA levels and histone acetylation of several genes, including caveolin-1. Consequently, the FABP7-ACLY interaction is associated with caveolin-1 transcription.

## Materials and Methods

### Antibodies and Reagents

Primary antibodies used in this study were rabbit polyclonal anti-mouse FABP7 established in our laboratory [21], rat monoclonal anti-GFAP (Thermo Fisher Scientific Inc., MA, USA, Cat. No. 13-0300), rabbit polyclonal anti-GAPDH (Santa Cruz, TX, USA, Cat. No. sc-25778), rabbit polyclonal anti-caveolin-1 (Santa Cruz, Cat. No. sc-894), rabbit polyclonal anti-Histone H3 (Abcam, Cambridge, England, Cat. No. ab1791), mouse monoclonal anti-EGFR (Santa Cruz, Cat. No. sc-373746), mouse monoclonal anti-β-actin (Santa Cruz, Cat. No. sc-47778), mouse monoclonal anti-H3K27ac and -H3K27me3 for CHIP assay (generous gift from Dr. Kimura) [31], total OXPHOS rodent WB antibody cocktail (Abcam, Cat. No. ab110413), rabbit polyclonal anti-acetyl lysine (Abcam, Cat. No. ab80178), rabbit polyclonal anti-H3K27ac for western blot (Abcam, Cat. No. ab4729), rabbit polyclonal anti-H3K9ac (Merck Millipore, MA, USA, Cat. No. 07-352), rabbit monoclonal anti-H4K16ac (Abcam, Cat. No. ab109463), rabbit monoclonal anti-H4 (acetyl K5, K8, K12, K16) (Abcam, Cat. No. ab177790), rabbit polyclonal Histone H4 (Abcam, Cat. No. ab10158), rabbit monoclonal anti-ACLY (Abcam, Cat. No.ab40793), mouse monoclonal anti-GST (Wako, Osaka, Japan, Cat. No. 017-21854), goat polyclonal anti-FABP7 (Santa Cruz, Cat. No. sc-16056), and anti-GLAST (ACDS-1)-APC conjugate (Milteny Biotec, Bergisch Gladbach, Germany, Cat. No. 130-098-803). Second antibodies used in this study were goat anti-rabbit IgG (H+L) Alexa Fluor 488 conjugated (Thermo Fisher Scientific Inc., Cat. No. A27034), goat anti-rat IgG (H+L) Alexa Fluor 594 conjugated (Thermo Fisher Scientific Inc., Cat. No. A-11007), goat anti-rabbit IgG (H+L) Alexa Fluor 594 conjugated (Thermo Fisher Scientific Inc., Cat. No. A-11012), goat anti-rabbit IgG (H+L) HRP conjugated (Merck Millipore, Cat. No. AP307P), goat anti-mouse IgG HRP conjugated (Merck Millipore, Cat. No. AP124P) and biotinylated rabbit anti-goat IgG (Vector Laboratory, CA, USA, Cat. No. BA-5000). DAPI (4′,6-diamidino-2-phenylindole, dihydrochloride), Hoechst®33342 and MitoTracker™ Green FM were purchased from Thermo Fisher Scientific (Cat. Nos. D1306, 62249, and M7514, respectively).

### Animals

The generation of FABP7 gene knockout mice was described previously [32]. C57BL/6 (WT) and FABP7-KO mice of same genetic background (FABP7-KO) were used in this study. Mice were fed standard chow and maintained under specific pathogen-free conditions. All experimental protocols were reviewed by the Ethics Committee for Animal Experimentation of Tohoku University Graduate School of Medicine and carried out according to the Guidelines for Animal Experimentation of the Tohoku University Graduate School of Medicine and under the law and notification requirements of the Japanese government.

### Cells and Transfection

Primary astrocytes were prepared from cerebral cortices of 0–1-day-old WT and FABP7-KO mice, as described previously [23]. In brief, following isolation of cortices and removal of the meninges, olfactory bulb, and hippocampus, dissociated cells were treated with 2.5% (w/v) trypsin (Thermo Fisher Scientific Inc.) for 10 min. Cells were resuspended in Dulbecco’s modified Eagle’s medium (DMEM, Thermo Fisher Scientific Inc.) containing 10% (v/v) heat-inactivated fetal bovine serum (FBS) (Thermo Fisher Scientific Inc.) and 1% (v/v) penicillin/streptomycin (Thermo Fisher Scientific Inc.), and filtered through a 100-μm cell strainer (BD Falcon, NJ, USA). Finally, cells were seeded in T75 flasks (BD Falcon) at a density of 2 × 10^7^ cells. Medium was replaced every third day. After 7–9 days in vitro, culture flasks were shaken for 24 h at 200 rpm to remove microglia and oligodendrocyte progenitor cells. The remaining astrocytes on the adherent monolayer were detached with 0.05% (w/v) trypsin (Thermo Fisher Scientific Inc.) and 0.02% (w/v) EDTA (Sigma-Aldrich Japan, Tokyo, Japan) and seeded into appropriate plates and dishes and grown for 6–7 days until confluent. The purity of astrocytes was confirmed to be > 95% by GFAP immunostaining. Primary cultured astrocytes for both genotypes were isolated, passaged, and analyzed at the same time to minimize bias.

The mouse embryonic fibroblast cell line NIH-3T3, the mouse astrocytoma cell line KR158, and HEK293T cells were obtained from Yamaguchi University Center for Gene Research. They were maintained by passage in DMEM containing FBS and 1% (v/v) penicillin/streptomycin. The constructed vectors were transfected into the cell line using Lipofectamine® LTX Reagent with PLUS™ Reagent (Thermo Fisher Scientific Inc.) following the manufacturer’s instructions. Cells were used for experiments 48 h after transfection.

### Construction of Expression Vectors

The coding region of mouse Fabp7 was amplified by PCR and amplified cDNA was subcloned into the pcDNA ™3.1^(+)^ mammalian expression vector (Thermo Fisher Scientific Inc.). The sequence of nuclear localization signal peptide (NLS, CCA AAG AAG AAG CGA AAG ATG) and nuclear export signal peptide (NES, AGT CTG GCA GCT GAG TTC CGA CAC CTG CAA CTG AAG GAA) were reported previously [33, 34]. Primer list to obtain amplified cDNA for Fabp7 with NLS at N or C terminus was shown in Supplemental Table 1. Amplified cDNA for Fabp7 with NES at N or C terminus was synthesized by GENEWIZ, Inc. (NJ, USA). Each of amplified cDNA was subcloned into pcDNA ™3.1^(+)^ mammalian expression vector.

A FLAG epitope tag was inserted into pCAGGS expression vector kindly gifted by Dr. Miyazaki [35]. Amplified mouse Fabp7 cDNA was subcloned into pCAGGS-FLAG vector to obtain Fabp7 with FLAG tag at N or C terminus.

Full-length caveoli-1 promoter (*Cav1* (− 1348/− 1)) was generated by PCR and subcloned into pGL3-basic vector (Promega, WI, USA). For construction of *Cav1* promoter deletion mutants, amplified PCR products of *Cav1* (− 1348/− 1) were treated with the following restriction enzyme: HindIII (*Cav1* (− 726, − 1)), EcoRI (*Cav1* (− 461, − 1)), XhoI (*Cav1* (− 315, − 1)), and BglII (*Cav1* (− 74, − 1)), and subcloned into pGL3-basic vector. To construct the mutation in *Cav1* (− 233, − 229), (− 116, − 110), and (− 108, − 102) on *Cav1* promoter, QuikChange Site-Directed Mutagenesis kit (Agilent Technologies; CA, USA) was used following the manufacturer’s instructions. For the amplification of *Cav1* promoter region including the mutation, primers showed in Supplemental Table 1 were used.

### Tet-Induced Stable Expression

Doxycycline-induced FABP7 expression system using lentivirus was prepared as below. pCW-FABP7, -FABP7-NLS, -FABP7-NES, or -control vector were transfected into HEK293T cells with pCAG-HIVgp and pCMV-VSV-G-RSV-Rev vector using lipofectamine 2000 (Thermo Fisher Scientific Inc.). The next day, the culture medium was replaced with fresh medium. Forty-eight hours after the medium change, the viral supernatants were collected and filtered. NIH-3T3 cells were suspended in the medium containing the virus, and rotated at room temperature for 1 h with 10 μg/ml hexadimethrine bromide (Sigma-Aldrich Japan). More than 1 week after infection, FABP7-expressing cells, which were venus-positive, were collected using a flow cytometer FACS Aria II (BD bioscience, NJ, USA). Each sorted cell was seeded in the plate and venus-positive expression was confirmed by confocal scanning-laser microscopy (Zeiss LSM780 META, Carl Zeiss, Oberkochen, Germany).

### Quantitative Real-Time PCR

Total RNA was extracted using an RNeasy Plus Mini Kit (Qiagen, Netherlands). Total RNA (4 μg) was reverse transcribed using anchored-oligo (dt)18 primers (Transcriptor High Fidelity cDNA Synthesis Kit; Roche, Basel, Switzerland). Quantitative real-time PCR (qPCR) was performed in an Applied Biosystems StepOnePlus™ real-time PCR system (Thermo Fisher Scientific Inc.) using TaqMan probes. The following mouse-specific TaqMan® probes were used: Mm01253033_m1 for *Gfap*, Mm00445225_m1 for *Fabp7*, Mm00483057_m1 for *Cav1*, Mm01129337_g1 for *Cav2*, Mm00600697_m1 for *Slc1a3* (Glast), Mm01248771_m1 for *Rbfox3* (NeuN), Mm01266402_m1 for *Mbp*, Mm00434455_m1 for *Itgam* (Cd11b), Mm99999915_g1 for *Gapdh*, Mm02619580_g1 for *Actb*, Mm03928990_g1 for *Rn18s*, Mm00434764_m1 for *Lpl*, Mm00453002_m1 for *Scpep1*, and Mm01187858_m1 for *Egfr*. Quantification was performed by normalizing cycle threshold (Ct) values to *Gapdh* and analyzed by the comparative Ct method with Applied Biosystems StepOnePlus™ real-time PCR system software v2.0 (Thermo Fisher Scientific Inc.).

### Western Blot

Tissue and cell lysates were prepared in sodium dodecyl sulfate polyacrylamide gel electrophoresis (SDS-PAGE) sample buffer containing protease and phosphatase inhibitors (Roche). Subcellular protein from primary astrocytes was obtained using a Subcellular Protein Fractionation Kit for Cultured Cells (Thermo Fisher Scientific Inc.). Protein concentrations were determined by BCA assay kit (Thermo Fisher Scientific Inc.). The lysates were resolved by SDS-PAGE and transferred to a polyvinylidene difluoride membrane (Merck Millipore). The membrane was blocked with 0.1% (v/v) Tween 20 and 5% (w/v) bovine serum albumin (Wako) in PBS, and incubated with primary antibody overnight at 4 °C followed by incubation with secondary antibody. Detection was performed with the ECL Western Blot Detection Kit (Thermo Fisher Scientific Inc.). Coomassie brilliant blue (CBB) staining showed that the proteins were equally isolated and loaded in SDS-PAGE.

### Immunohistochemistry and Immunocytochemistry

Immunohistochemistry was performed as described previously [36]. Briefly, the mice were perfused intracardially with 4% paraformaldehyde (PFA; Nacalai Tesque, Kyoto, Japan) under anesthesia. The brain was sampled and post-fixed with fresh 4% PFA for overnight at 4 °C. For frozen section, samples were placed in graded concentrations of sucrose solution for cryoprotection. Coronal sections (20 μm) were sliced using a cryostat (CM1850; Leica, Nussloch, Germany) in accordance with a mouse brain atlas. Brain sections were incubated overnight at 4 °C with first antibodies. The sections were then incubated with secondary antibodies. After nuclear staining with DAPI, slides were coverslipped using Fluoromount (Diagnostic BioSystems, Pleasanton, CA). Samples were examined by confocal scanning laser microscopy.

For immunocytochemistry, culture dishes containing cells were washed with D-PBS(−) twice and fixed with 4% PFA. Fixed cells were permeabilized with 0.3% (v/v) Triton X-100 in PBS and blocked with FBS in PBS. The reaction with primary antibodies was performed overnight at 4 °C, and the reaction with secondary antibodies and DAPI was performed for 1 h at room temperature. Samples were examined by confocal scanning laser microscopy.

### Luciferase Assay

NIH-3T3 cells were seeded into 24-well plate the day before transfection at a density of 5 × 10^4^ cells in DMEM with 10% FBS without antibiotics. pcDNA3-mFABP7, pGL3-*Cav1*-promoter-luc, and pTk-Renilla-luc were co-transfected at the ratio of 3:3:1. Co-transfectant was mixed with lipofectamine at a ratio of 4:1 (μl:μg). For pGL3-*Cav1*-promoter-luc, pGL3-*Cav1* (− 1384, − 1), (− 722, − 1), (− 458, − 1), (− 313, − 1), (− 209, − 1), (− 74, − 1) were used separately, and pTk-Renilla-luc was used for an internal standard for transfection efficiency. After 6 h incubation, medium containing lipofectamine and DNA were removed and replaced with DMEM with 10% FBS and antibiotics. 48 h after transfection, luciferase activity was evaluated using Dual-Luciferase^®^ reporter assay system (Promega) following the manufacturer’s instructions. Relative light units (RLUs) were determined by Flex station 3 microplate reader (Molecular Devices, CA, USA). Each experiment was performed 3 times and the results are represented as mean ± SEM.

### Bisulfite Genomic Sequence

Bisulfite genomic sequencing was performed as reported previously [37]. In brief, genomic DNA was extracted using High Pure PCR Template Preparation Kit (Roche) following the manufacturer’s instructions. The bisulfite reaction was carried out using EpiTect^®^ Plus DNA Bisulfite Kit (Qiagen) following the manufacturer’s instructions. The DNA fragments covering the transcriptional regulatory region of caveolin-1 (− 346 to + 71) were amplified by PCR using the set of primers as shown in Supplemental Table 1. The PCR conditions were 95 °C for 10 min, and 38 cycles of 95 °C for 30 s, 60 °C for 30 s, and 72 °C for 30 s, with a final extension at 72 °C for 10 min. The resulting products were subjected to agarose gel electrophoresis and purified using a Wizard® SV Gel and PCR Clean-Up System (Promega). The PCR products were cloned into pGEM-T easy vector (Promega), and ten or more transformed colonies from each of two independent PCRs were sequenced to determine the methylation status. Sequencing was performed using an Applied Biosystems 3730xl DNA Analyzer with Applied Biosystems Big Dye Terminator V3.1 (Thermo Fisher Scientific Inc.). The software QUMA (quantification tool for methylation analysis) was used for analysis.

### Chromatin Immunoprecipitation Assay

Cells were cross-linked by addition of formaldehyde into the medium at a final concentration of 1% and incubated for 10 min at 37 °C. Cross-linking was terminated by addition of glycine (0.125 M, final concentration). Cells were washed with ice-cold PBS containing protease inhibitor (Roche) and resuspended in 1% SDS lysis buffer with protease inhibitor. The lysates were sonicated using a Bioruptor ultrasonicator (Cosmo-bio; Tokyo, Japan). The sonicated lysates were then diluted with ChIP dilution buffer (0.01% SDS, 1.1% Triton X-100, 1.2 mM EDTA, 16.7 mM Tris-HCl (pH 8.0), 167 mM NaCl, with protease inhibitors). Ten percent of the supernatant was kept as input controls. The chromatin was incubated with antibodies for acetylated H3K27 and trimethylated H3K27 at 4 °C overnight. Anti-normal mouse IgG antibody was used as a negative control. Immune complexes were collected with 40 μl of Dynabeads Protein A (Thermo Fisher Scientific Inc.) and washed once for 5 min on a rotating platform with 1 ml each of the following buffers in sequence: low-salt wash buffer (0.1% SDS, 1% Triton X-100, 2 mM EDTA, 20 mM Tris-HCl (pH 8.0), 150 mM NaCl), high-salt wash buffer (0.1% SDS, 1% Triton X-100, 2 mM EDTA, 20 mM Tris-HCl (pH 8.0), 1500 mM NaCl), LiCl wash buffer (250 mM LiCl, 1% Nonidet P-40, 1% sodium deoxycholate, 1 mM EDTA, 10 mM Tris-HCl (pH 8.0), and twice with TE (10 mM Tris-HCl (pH 8.0), 1 mM EDTA)). Immune complexes were eluted with 250 μl elution buffer (1% SDS, 0.1 M NaHCO3, 10 mM dithiothreitol). Cross-linking of the immunoprecipitated chromatin complexes and input controls (10% of the total soluble chromatin) was reversed by heating the samples at 65 °C overnight and subjected to proteinase K treatment. The DNA was purified using a QIAquick PCR purification kit (Qiagen) following the manufacturer’s instructions. The relative levels of histone modifications of each target sequence were analyzed by qPCR using SYBR® Premix Ex Taq™ II (Takara, Tokyo, Japan) and Light Cycler 1.5® Carousel-based system (Roche). The list of primers to amplify the several promoter regions for *Cav1*, *Lpl*, *Scpep1*, *Cav2*, and *Egfr* is shown in Supplemental Table 1.

### Immunoprecipitation

Cells were washed with ice-cold PBS containing protease inhibitor (Roche) and resuspended in sonication buffer (50 mM Tris-HCl (pH 8.0, 150 mM sodium chloride, 1 mM EDTA, 1 mM PMSF, 1 mM DTT, 1 mg/ml lysozyme)) and sonicated with an ultrasonic homogenizer. Cell lysates were mixed with nProtein A Sepharose™ 4 Fast Flow (GE Healthcare Life Science; Little Chalfont, England) conjugated with 5% BSA in dilution buffer (16.7 mMTris-HCl (pH 8.0), 150 mM sodium chloride, 1 mM EDTA) in advance, and incubated at 4 °C for 4 h and centrifuged to eliminate the nonspecific binding of proteins. After centrifugation, 10% of the supernatant was kept as input controls. The supernatant was retained and incubated with anti-DYKDDDDK (FLAG) tag antibody magnetic beads (Wako) at 4 °C for overnight. After incubation, magnet beads with immunocomplex were washed with dilution buffer and immunocomplex was removed from magnetic beads by competitive elution method using FLAG peptides (Wako). Supernatant containing immunocomplex was resolved by SDS-PAGE and gels were stained using silver staining kit for mass spectrometry (APRO Science, Tokushima, Japan).

### Mass Spectrometry Analysis

The bands developed by silver staining were excised and subjected to a trypsin in-gel digestion procedure. After overnight tryptic digestion, the resulting peptides in the gel blocks were extracted and one-half of each sample was subjected to LC-MS/MS using an LTQ Orbitrap Velos mass spectrometer equipped with an EASY nanoLC 1000 system (Thermo Fisher Scientific Inc.). The peptides were separated on a PepMap C18 column (75 μm × 25 cm, Thermo Fisher Scientific Inc.) using a linear gradient generated by solution A (0.1% formic acid in water) and B (0.1% formic acid in acetonitrile): 2% B to 22% B in 36 min, to 40% B in 39 min, and then to 95% B in 41 min. The data acquisition of every sample was done for 48 min after the LC gradient was started, where MS1 scans from m/z = 321 to 1600 were carried out in the orbitrap with the resolution set at 60,000 with a lock mass at m/z = 445.120025, followed by top-15 MS2 acquisition by collision-induced dissociation in the ion trap in the normal resolution mode. The raw data files derived from samples in the same SDS-PAGE lane were converted together into a single MASCOT generic format file and were used for the database search by MASCOT (version 2.5.1, Matrix Science) against the mouse proteins in Swissprot and TrEMBL (July 2016), and a custom database including contaminant proteins. The peptide expectation value cutoff was set at 0.05. Carbamidomethylated cysteine as a fixed modification and protein N-terminal acetylation and oxidation of methionine as possible variable modifications were considered in the search. The false discovery rates were automatically adjusted to 1% by MASCOT percolator in every search.

### Functional Nuclear Isolation

Functional nuclear isolation was performed using commercially available nuclei isolation kit (Nuclei Pure Prep Isolation Kit; Sigma-Aldrich Japan) as reported previously [6]. In brief, adherent cells were washed with D-PBS and scraped from the plate in the presence of lysis buffer. Cells in lysis media were carefully placed on the top of a 1.8 M sucrose gradient and the resulting suspension was centrifuged at 30,000×*g* for 45 min in a precooled swinging bucket ultracentrifuge (Optima XPN-80; Beckman Coulter Inc.CA, USA). Nuclei at the bottom of the centrifuge were washed with buffer provided with the kit. Purity of nuclei was assessed by Hoechst® 33342 and MitoTracker imaging and western blot using anti-OXPHOS, anti-GAPDH, and anti-total histone antibodies. For functional experiments, isolated nuclei were used immediately.

### Acetyl-CoA Measurement

Acetyl-CoA measurement was performed using commercially available kit (PicoProbe Acetyl-CoA Assay kit (Fluorometric); Abcam). In brief, whole cells or isolated nuclei were resuspended in assay buffer provided with the kit, and homogenized with dounce homogenizer in ice box. Nuclear lysis was reacted with kit solution following the manufacturer’s instructions, and fluorescence was detected by Flex station 3 microplate reader (excitation/emission: 535/587 nm).

### Recombinant Proteins

Mouse Fabp7 cDNA was subcloned into the bacterial expression vector pGEX-6p-3 (GE Healthcare Life Science), and transformed into *Escherichia coli* BL21 (DE3). The *E.coli* cells were grown at 37 °C and induced at an OD_600_ of 1.0, with 0.1 mM isopropyl thiogalactoside for 6 h at 20 °C. The pFastBac1-GST vector was constructed by inserting the glutathione S-transferase (GST) coding sequence at the 5′ terminus to the multiple cloning site of pFastBac1 (Thermo Fisher Scientific Inc.) as reported previously [38]. The PCR-amplified cDNA fragments of full-length mouse Fabp7 (FABP7wt) and point mutation (R126A/Y128A) (FABP7mut) were subcloned into pFastBac1-GST vectors. GST-FABP7wt and GST-FABP7mut were expressed in Sf21 cells using the Bac-to-Bac baculovirus expression system (Thermo Fisher Scientific Inc.). The harvested cells were lysed by ultrasonicator, and the centrifuged supernatant was added to a 50% slurry of Glutathione Sepharose 4B (GE Healthcare Life Science) pre-equilibrated with PBS (pH 7.3) and incubated for 2 h. After extensive washing with PBS, GST-fusion proteins were eluted by competitive elution using reduced GST (Wako). Protein solutions were concentrated with Amicon Ultra-0.5 ml centrifugal filter devices (Merck Millipore) with a 30-kDa molecular weight cutoff and dilapidated by incubation with Lipidex-1000 (PerkinElmer, Waltham, MA, USA) as reported previously [39, 40]. Protein purity was confirmed by CBB staining following SDS-PAGE, and the protein concentrations were determined using BCA assay kit.

### ANS Binding Assay

Binding assay using the fluorescent probe, 1-anilinonaphthalene-8-sulfonic acid (ANS) (Cayman Chemical, MI, USA), was based on a procedure as reported previously [41]. Titrations of recombinant proteins with ANS were studied by measuring changes in fluorescence during titrations of ANS into a fixed concentration of recombinant proteins. The mixtures of ANS and each recombinant protein (0.8 mM) in 50 ml buffer (10 mM potassium phosphate, 1.62 mM disodium hydrogen phosphate, 2.74 mM NaCl, and 40.54 mM KCl, pH 7.4) were kept at 25 °C for 3 min in the dark. Fluorescence was measured using Flex station 3 microplate reader (excitation/emission: 355/460 nm).

### GST Pull-Down Assay

GST or GST-FABP7 expressed in Sf21 cells were immobilized on Glutathione Sepharose 4B. The beads were incubated at 4 °C for 4 h in cell lysis which were prepared in the same protocol as for immunoprecipitation assay. The beads were washed three times with lysis buffer and boiled in sample buffer. Samples were analyzed by western blot.

### ACLY Activity Assay

ACLY activity was measured by malate dehydrogenase coupled method as reported previously [42, 43] with slight modifications. Cell lysates with recombinant proteins were added at a 1:19 ratio to the reaction mixture containing 100 mM Tris-HCl (pH 8.7), 20 mM potassium citrate, 10 mM MgCl_2_, 10 mM DTT, 0.5 U/ml malate dehydrogenase, 0.33 mM CoASH, 0.14 mM NADH, and 5 mM ATP (all from Sigma-Aldrich Japan). Change in absorbance at 340 nm was read every 15 s over 35 min in microplate reader. Change in absorbance in the absence of exogenous ATP was subtracted from change in the presence of ATP and the result was normalized to protein concentration to determine the specific ACLY activity.

### Human Gene Expression Profiling

Gene expression was assessed through a web-based tool (GEPIA) as shown previously [44]. Provisional datasets from TCGA and GTEx consortiums were used for the analysis.

### Statistical Analysis

All data represent the mean ± s.e.m. of at least three independent experiments. Statistical comparisons of means were made by Student’s two-tailed unpaired *t* test or one-way ANOVA followed by the Tukey test for multiple comparisons. *P* values less than 0.05 were considered statistically significant. Analysis was performed using the Microsoft Excel.

## Results

### FABP7 Localizes in Nucleus and Cytoplasm, and Regulates Caveolin-1 Expression

**Fig. 1:**
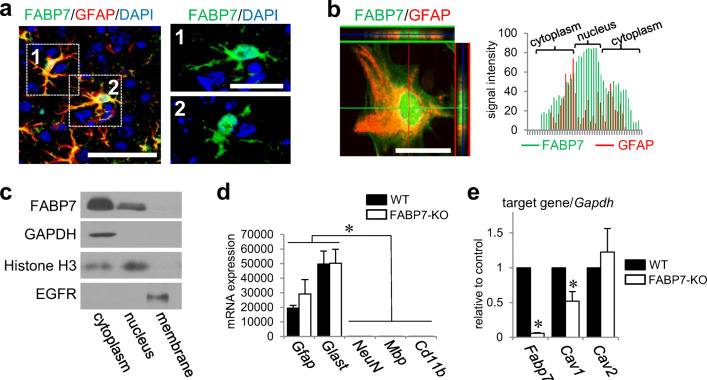
FABP7 localizes in nucleus and cytoplasm and regulates caveolin-1 expression. **a** Co-immunofluorescence staining of FABP7 (green), GFAP (red), and DAPI (blue) in sectioned prefrontal cortex of mouse brain. Right images show the high-magnification image for each cropped cells. Scale bar: 100 μm (left), 50 μm (right). **b** Immunofluorescence staining of FABP7 (green) and DAPI (blue) in primary cultured astrocytes and observed by confocal laser scanning microscopy. The red and green orthogonal projection lines through the central position of the nucleus indicating co-localization denote the different planes (red-right panel and green-upper panel) reconstructed from the Z-plane cross sections. Scale bar: 50 μm. **c** Western blot for FABP7 protein expression in cellular fraction from cultured astrocytes. GAPDH, histone H3, and EGFR are used as the marker of cytoplasm, nucleus, and membrane, respectively. **d** qPCR analysis for mRNA expression of *Gfap*, *Glast*, *NeuN*, *Mbp*, and *Cd11b* for confirmation of the purity of sorted astrocytes from mouse prefrontal cortex. *Gfap* and *Glast* are used as a marker of astrocyte, and *NeuN*, *Mbp*, and *Cd11b* are used as a marker of neuron, oligodendrocyte, and microglia, respectively. **e** qPCR analysis for mRNA expression of *Fabp7*, *Cav1*, and *Cav2* in sorted astrocytes from prefrontal cortex of WT and FABP7-KO mice. Each target gene level was calculated relative to WT as control. Data shown are the means ± s.e.m. and representative of 3 independent experiments. *p < 0.05 versus WT

We used immunohistochemistry to examine FABP7 cellular localization in mouse cortices. Our results showed that FABP7 was expressed in the nucleus and cytoplasm (Fig. 1a). FABP7 was similarly localized in primary cultured astrocytes, according to immunocytochemistry and western blots (Fig. 1b, c; S1a and S1b), and notably high expression of FABP7 is observed in nuclei (Fig. 1b). Confirming our previous findings, FABP7 deficiency alters caveolin-1 gene and protein expression in different cells, including sorted astrocytes from mouse cortex (Fig. 1d, e; S1f), as well as primary cultured astrocytes (Fig. S1c–e).

### FABP7 Affects Caveolin-1 Promoter Activity to Regulate Caveolin-1 Expression

**Fig. 2:**
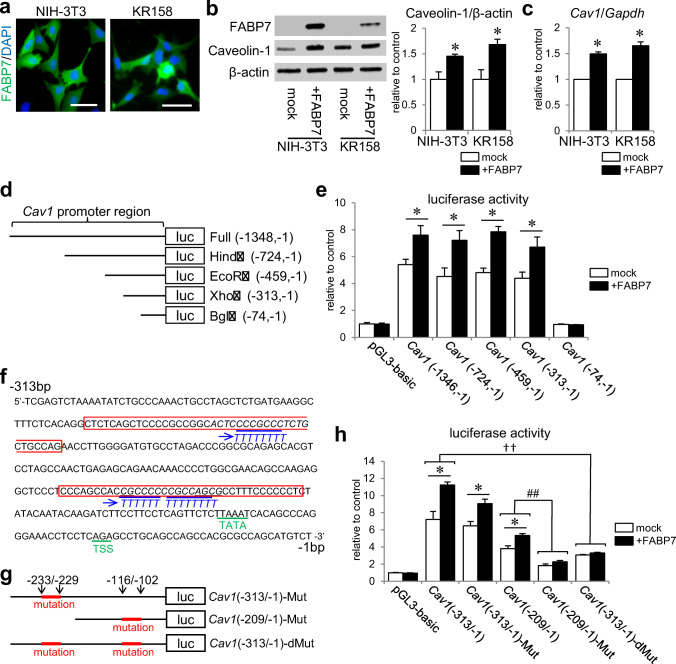
FABP7 affects caveolin-1 promoter activity to regulate caveolin-1 expression. **a** Immunofluorescence staining of FABP7 (green), and DAPI (blue) in NIH-3T3 cells and KR158 cells. Scale bar: 50 μm. **b** Western blot for FABP7 and caveolin-1 protein expression in NIH-3T3 cells and KR158 cells. Bar graph shows band density analyzed using NIH-Image J. **c** qPCR analysis for mRNA expression of *Cav1*, *Lpl*, *Scpep1*, *Cav2*, and *Egfr* in NIH-3T3 cells transfected with mock, FABP7, FABP7-NLS (N terminus), and FABP7-NES (N terminus). **d** Schematic representation of luciferase reporter vectors containing full-length *Cav1* promoter, and 5′ deletion mutants using different restriction enzymes. **e** Luciferase activity assay in NIH-3T3 cell with or without FABP7 overexpression, co-transfected with different reporter vectors of *Cav1* promoter. Activity was calculated relative to cells transfected with pGL3-basic luciferase vector. **f** DNA sequence of *Cav1* promoter between – 313-bp and – 1-bp upstream of start codon showing the CG-rich regions. **g** Schematic representation of luciferase reporter vectors containing mutated *Cav1* (− 313/− 1), mutated *Cav-1* (− 209/− 1), and double mutations in *Cav1* (− 313/− 1). **h** Luciferase activity assay in NIH3T3 cell with or without FABP7 expression, co-transfected with indicated *Cav1* luciferase vectors. Activity was calculated relative to cells transfected with pGL3-basic luciferase vector. Data shown are the means ± s.e.m. and representative of 3 independent experiments. *p < 0.05 versus mock. For panel **h**, * < 0.05 between mock and NIH-3T3, †† < 0.01, between *Cav1* (− 313/− 1) and *Cav1* (− 313/− 1) double mutation in both mock and NIH-3T3 with FABP7, ## < 0.01 between *Cav1* (− 209/− 1) and *Cav1* (− 209/− 1) mutation in both mock and NIH-3T3 with FABP7

To determine the role of FABP7, we constructed an FABP7 overexpression system using NIH-3T3 and KR158 cells (Fig. 2a, b). Overexpressing FABP7 increased caveolin-1 expression at protein and transcriptional levels (Fig. 2b, c). We then performed a luciferase reporter assay to identify the caveolin-1 promoter region on which FABP7 acts. A previous study indicated that several transcription factors regulate caveolin-1 promoter activity, and that they bind to the region between – 1200- and – 91-bp upstream from *Cav1* start codon [26]. As such, we constructed a *Cav1* (− 1348/− 1) luciferase reporter vector and its 5′ deletion mutants using several restriction enzymes (Fig. 2d). Each vector was transiently transfected into NIH-3T3 cells with or without FABP7 expression vectors. Luciferase activity was almost equally elevated in mock cells of *Cav1* (− 1348/− 1), *Cav1* (− 724/− 1), *Cav1* (− 459/− 1), and *Cav1* (− 313/− 1), at around 5-fold induction compared with the control pGL3-basic vector (Fig. 2e). However, activity was drastically reduced in *Cav1* (− 74/− 1) (Fig. 2e), indicating that the promoter region between − 313- and – 74-bp upstream from the start codon is an essential element for *Cav1* transcription. When these vectors were co-transfected with FABP7 expression vector, *Cav1* (− 1348/− 1), *Cav1* (− 724/− 1), *Cav1* (− 459/− 1), and *Cav1* (− 313/− 1) were significantly activated compared with mock cells (~ 7–8-fold induction) (Fig. 2e). However, luciferase activity in *Cav1* (− 74/− 1) was low even with FABP7 expression (Fig. 2e). Thus, FABP7 involvement in *Cav1* transcription occurs through the *Cav1* promoter (between − 313 and − 74 bp). Several transcription factors regulate a GC-rich element in this region [26]. This element is also epigenetically regulated through DNA methylation and histone acetylation [29, 45] (Fig. 2f).

To examine FABP7 involvement in modifying *Cav1* promoter activity through the two GC-rich regions, we performed luciferase assays on the following *Cav1-*GC mutation vectors: *Cav1* (− 313/− 1)-Mut, *Cav1* (− 209/− 1)-Mut, and *Cav1* (− 313/− 1)-dMut (Fig. 2f, g). *Cav1* (− 313/− 1)-Mut and *Cav1* (− 313/− 1) did not differ in amplitude for both mock and FABP7-overexpressed cells (Fig. 2h). In contrast, luciferase activation in *Cav1* (− 209/− 1)-Mut and *Cav1* (− 313/− 1)-dMut decreased in mock and FABP7-overexpressed cells. Additionally, the significant difference in amplitude between mock cells and FABP7-overexpressed cells was eliminated (Fig. 2h). These results suggest that FABP7 is involved in the modification of caveolin-1 promoter activity through the GC-rich region between − 209 and − 74 bp.

### FABP7 Regulates Caveolin-1 Expression Through Acetylation of Histone-H3 Lysine-27 on Caveolin-1 Promoter

**Fig. 3:**
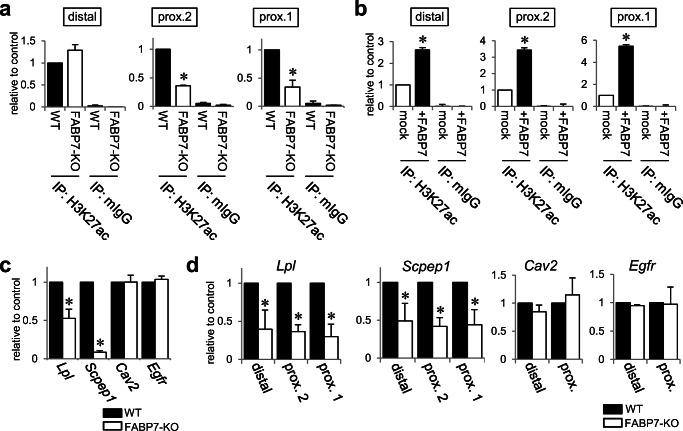
FABP7 regulates caveolin-1 expression through acetylation of histone-H3 lysine-27 on caveolin-1 promoter. **a**, **b** ChIP assays and subsequent qPCR for H3K27ac on each caveolin-1 promoter region of WT or FABP7-KO primary cultured astrocytes (**a**), and mock or FABP7-overexpressed NIH-3T3 cells (**b**). Mouse IgG was used for negative control. **C** qPCR analysis for mRNA expression of *Cav1*, *Lpl*, *Scpep1*, *Cav2*, and *Egfr* in WT and FABP7-KO astrocytes. **d** ChIP assays and subsequent qPCR with proximal-1 primer set of *Cav1*, *Lpl*, *Scpep1*, *Cav2*, and *Egfr* for the levels of H3K27ac in WT and FABP7-KO astrocytes. Data shown are the means ± s.e.m. and representative of 3 independent experiments. *p < 0.05 versus WT or mock

We used CHIP assay and quantitative RT-PCR to examine acetylation and trimethylation of histone-H3 lysine-27 (H3K27ac and H3K27me3) in three *Cav1* promoter regions (Fig. S2a). In FABP7-KO astrocytes, H3K27ac levels in the two proximal regions decreased compared with WT astrocytes (Fig. 3a), while H3K27me3 levels increased (Fig. S2b). Furthermore, we demonstrated that among FABP7-overexpressed NIH-3T3 cells, H3K27ac levels increased in every promoter region compared with mock cells (Fig. 3b), but H3K27me3 levels remained unchanged (Fig. S2c). Additionally, we examined DNA methylation in *Cav1* promoter using bisulfite sequence analysis and revealed that *Cav1* promoter in mock NIH-3T3 cells contains several methylated CG elements (Fig. S3a). Both FABP7-overexpressed cells and negative control (mock + 5-Aza treatment) showed hypo-methylated CG elements (Fig. S3b), while *Cav1* promoter of FABP7-KO astrocytes contained more methylated CG elements than WT astrocytes (Fig. S3c). All these results suggest that DNA methylation in *Cav1* promoter is altered depending on FABP7 expression levels.

Using comparative DNA microarray analysis, we focused on candidate genes (serine carboxypeptidase1, *Scpep1*; lipoprotein lipase, *Lpl*) that are potentially regulated by FABP7 (Table 1) based on their promoter sequences. Quantitative RT-PCR confirmed that FABP7-KO astrocytes had lower *Scpep1* and *Lpl* expression than WT astrocytes (Fig. 3c). FABP7-KO astrocytes also had lower H3K27ac and elevated H3K27me3 in every region of *Lpl* and *Scpep1* promoters (Fig. 3d;S2d). However, histone acetylation of other genes, including *Cav2* and *Egfr*, was unaffected (Fig. 3d; S2d), suggesting that astrocytic FABP7 specifically affects gene expression through histone modification.

**Table 1:** Microarray result (the list of gene downregulated by FABP7 deficiency in astrocytes)

Gene symbol	Fold change	*p* value	Gene name
Fabp7	0.00043	1.52E−10	Fatty acid binding protein 7, brain
Scpep1	0.13733	4.24E−08	Serine carboxypeptidase 1
Pop4	0.13878	3.30E−09	Processing of precursor 4, ribonuclease P/MRP family (*S. cerevisiae*)
Slc2a4	0.13990	1.36E−08	Solute carrier family 2 (facilitated glucose transporter), member 4
Wdfy1	0.17149	8.71E−08	WD repeat and FYVE domain containing 1
Lpl	0.19016	2.30E−04	Lipoprotein lipase
Plvap	0.22900	1.50E−04	Plasmalemma vesicle–associated protein
Irgm2	0.24645	1.59E−05	Immunity-related GTPase family M member 2
Fam154b	0.25515	1.45E−02	Family with sequence similarity 154, member B
Gatad2a	0.28325	1.57E−07	GATA zinc finger domain containing 2A
Coil	0.30704	5.02E−07	Coilin
Agfg2	0.31200	1.03E−05	ArfGAP with FG repeats 2
Vmn1r48	0.32430	1.38E−04	Vomeronasal 1 receptor 48
Zfp672	0.36473	1.59E−07	Zinc finger protein 672
Napa	0.36635	1.14E−05	N-Ethylmaleimide sensitive fusion protein attachment protein alpha
Cd59a	0.38746	5.57E−06	CD59a antigen
Lgals4	0.39180	1.08E−03	Lectin, galactose binding, soluble 4
Ndrg4	0.39226	5.01E−06	N-Myc downstream regulated gene 4
Zfp503	0.40874	6.51E−06	Zinc finger protein 503
Eml2	0.41099	1.11E−03	Echinoderm microtubule associated protein like 2
Pisd-ps3	0.42044	8.65E−05	Phosphatidylserine decarboxylase, pseudogene 3
S100a3	0.42260	2.68E−02	S100 calcium binding protein A3
Mrps12	0.42286	1.37E−06	Mitochondrial ribosomal protein S12
Tmem88	0.42343	3.43E−04	Transmembrane protein 88
Gadd45gip1	0.42344	4.92E−07	growth arrest and DNA-damage-inducible, gamma interacting protein 1
Cdk5rap1	0.42795	2.56E−03	CDK5 regulatory subunit associated protein 1
Dcx	0.43198	6.03E−03	Doublecortin
Sp8	0.43692	5.82E−03	Trans-acting transcription factor 8
Rgs22	0.44513	3.04E−02	Regulator of G protein signaling 22
Hebp2	0.44852	3.45E−05	Heme binding protein 2
Gdf15	0.45464	1.44E−04	Growth differentiation factor 15
Vtn	0.45624	4.73E−02	Vitronectin
Srl	0.47381	2.13E−02	Sarcalumenin
Dlx1	0.47564	2.88E−03	Distal-less homeobox 1

Shown are representative genes which were downregulated in FABP7-KO astrocytes

### Nuclear Localization of FABP7 Increases Caveolin-1 Expression Via Modification of Histone Acetylation

**Fig. 4:**
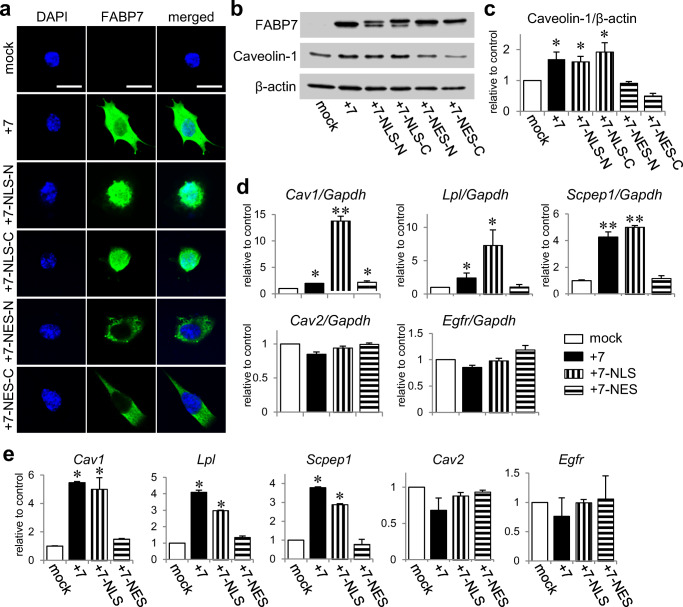
Nuclear localization of FABP7 increases caveolin-1 expression via modification of histone acetylation. **a** Immunofluorescence staining of FABP7 (green), and DAPI (blue) in NIH-3T3 cells transfected with mock, FABP7, FABP7-NLS (in C or N terminus), and FABP7-NES (in C or N terminus). Scale bar: 50 μm. **b, c** Western blot for FABP7 and caveolin-1 protein expression in NIH-3T3 cells transfected with mock, FABP7, FABP7-NLS (in C or N terminus), and FABP7-NES (in C or N terminus). Bar graph (**c**) shows band density analyzed using NIH-Image J. **d** qPCR analysis for mRNA expression of *Cav1*, *Lpl*, *Scpep1*, *Cav2*, and *Egfr* in NIH-3T3 cells transfected with mock, FABP7, FABP7-NLS (N terminus), and FABP7-NES (N terminus). **e** ChIP assays and subsequent qPCR with proximal-1 primer set of *Cav1*, *Lpl*, *Scpep1*, *Cav2*, and *Egfr* for the levels of H3K27ac in NIH3T3 cells transfected with mock, FABP7, FABP7-NLS (N terminus), and FABP7-NES (N terminus). Data shown are the means ± s.e.m. and representative of 3 independent experiments. *p < 0.05, **p < 0.01 versus mock

To gain mechanistic insight into the significance of nuclear localization of FABP7 in the epigenetic gene modification, we transfected FABP7 expression vectors in NIH-3T3 cells with either nuclear localization signal (NLS) peptides (FABP7-NLS) or nuclear export signal (NES) peptides (FABP7-NES) at FABP7 N or C termini. After immunocytochemical confirmation of FABP7 localization (Fig. 4a), we investigated whether altering FABP7 localization affected caveolin-1 expression. Western blot showed that caveolin-1 expression in FABP7-NLS-overexpressed cells (+ 7-NLS-N and –C) was increased at the same levels with FABP7 overexpressed cells (+ 7) (Fig. 4b, c), while caveolin-1 expression in FABP7-NES overexpressed cells (+ 7-NES-N and –C) was same with that in mock cells (Fig. 4b, c). Consistently, + 7-NLS significantly increased caveolin-1 mRNA expression compared with + 7 (Fig. 4d). These results suggest that nuclear FABP7 is highly involved in expression of the candidate genes.

Next, we investigated whether FABP7 localization affected H3K27ac and H3K27me3 levels in *Cav1*, *Lpl*, and *Scpep1* promoters*.* H3K27ac levels in + 7-NLS were same with that in + 7, but H3K27ac in + 7-NES was same with that of mock cells (Fig. 4e;S4a, S4c, and S4e). H3K27me3 levels were unchanged across all cell types (Fig. S4b, d, and f). Moreover, mRNA expression and histone acetylation/trimethylation of *Cav2* and *Egfr* did not change (Fig. 4d, e and S4g–j), suggesting that nuclear FABP7 specifically modulates gene expression through histone modification.

### FABP7 Deficiency in Astrocytes Decreased Specific Histone Lysine Acetylation and Acetyl-CoA in Cytoplasm and Nucleus

**Fig. 5:**
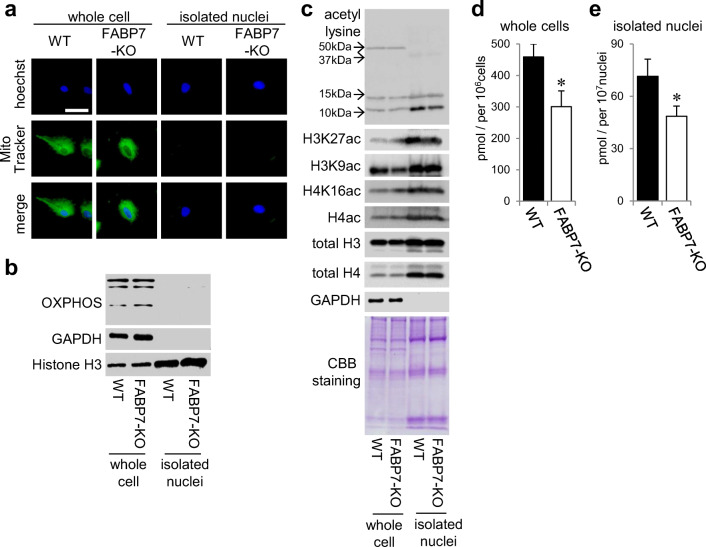
FABP7 deficiency in astrocytes decreased the levels of specific histone lysine acetylation and the levels of acetyl-CoA in cytoplasm and nucleus. **a** Imaging to confirm the purification of functional nuclei from primary cultured astrocytes. Hoechest33342 and MitoTracker are used as the marker of nuclei and mitochondria, respectively. **b** Western blot to confirm the purification of functional nuclei from primary cultured astrocytes. OXPHOS, GAPDH, and histone H3 are used as the marker of mitochondria, cytoplasm, and nuclei, respectively. **c** Western blot for acetyl lysine, H3K27ac, H3K9ac, H4K16ac, and H4ac in WT and FABP7-KO primary cultured astrocytes. **d**, **e** Quantitative analysis of acetyl-CoA in whole cells (**d**) and isolated functional nuclei (**e**) of primary cultured astrocytes. The levels were normalized by the number of cells or nuclei, respectively. Data shown are the means ± s.e.m. and representative of 3 independent experiments. *p < 0.05 versus WT

Because H3K27ac acetylation changed in the promoter of several tested genes, we evaluated other histone acetylation levels. For western blot analysis, we used protein from whole cells or isolated nuclei from primary cultured astrocytes. We confirmed purification of the latter through cell imaging with MitoTracker and Hoechst (Fig. 5a), and through western blot with anti-OXPHOS and histone antibodies (Fig. 5b). Interestingly, pan-acetyl lysine levels in approximately 10 kDa increased among isolated nuclei from WT astrocytes compared with FABP7-KO astrocytes (Fig. 5c). Furthermore, the levels of H3K27ac in isolated nuclei and H3K9ac in whole WT astrocytes were higher than those in FABP7-KO astrocytes, while H4ac acetylation did not differ between WT and FABP7-KO astrocytes (Fig. 5c), suggesting that FABP7 is involved in specific histone acetylation.

To elucidate further the mechanism of FABP7 involvement in histone acetylation, we focused on acetyl-CoA, the essential substrate for histone acetyltransferases (HATs). We evaluated whether FABP7 is involved in acetyl-CoA generation across separated subcellular compartments. Acetyl-CoA levels in whole cells and nuclei were lower in FABP7-KO astrocytes than in WT astrocytes (Fig. 5d, e), suggesting that FABP7 affects acetyl-CoA generation in both nuclear and cytoplasmic compartments.

### Nuclear Localization of FABP7 Increases Nuclear Acetyl-CoA Levels

**Fig. 6:**
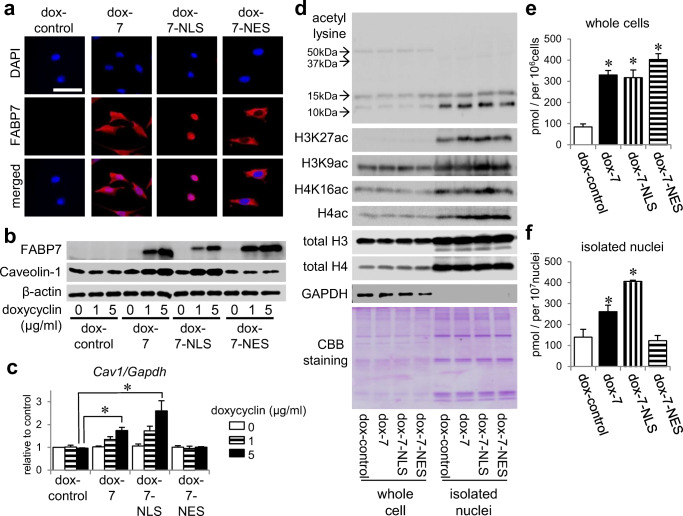
Nuclear localization of FABP7 increases nuclear acetyl-CoA levels. **a** Immunofluorescence staining of FABP7 (red) and DAPI (blue) in NIH-3T3 cells with doxycycline-induced control, FABP7, FABP7-NLS, and FABP7-NES. **b** Western blot for FABP7 and caveolin-1 expression in NIH-3T3 cells with doxycycline-induced control, FABP7, FABP7-NLS, and FABP7-NES. **c** qPCR analysis for mRNA expression of *Cav1* in NIH-3T3 cells with doxycycline-induced control, FABP7, FABP7-NLS, and FABP7-NES. **d** Western blot for acetyl lysine, H3K27ac, H3K9ac, H4K16ac, and H4ac in NIH-3T3 cells with doxycycline-induced control, FABP7, FABP7-NLS, and FABP7-NES. **e**, **f** Quantitative analysis of acetyl-CoA in whole cells (**e**) and isolated functional nuclei (**f**) of NIH-3T3 cells with doxycycline-induced control, ubiquitous FABP7, FABP7-NLS, and FABP7-NES. The levels were normalized by the number of cells or nuclei, respectively. Data shown are the means ± s.e.m. and representative of 3 independent experiments. *p < 0.05 versus dox-control

To elucidate how FABP7 regulates acetyl-CoA levels and histone acetylation, we constructed NIH-3T3 cells with doxycycline-inducible FABP7, FABP7-NLS, and FABP7-NES expression (Fig. 6a). Caveolin-1 expression was regulated by doxycycline-induced FABP7 expression in a dose-dependent manner (Fig. 6b, c). Consistent with the transient overexpression system (Fig. 4), caveolin-1 expression increased in FABP7-NLS-overexpressed cells to the same level as in FABP7 overexpressed cells, but did not change in FABP7-NES overexpressed cells (Fig. 6b, c). H3K27 acetylation was higher in FABP7- or FABP7-NLS-overexpressed cell nuclei than in control, but not in FABP7-NES overexpressed cell nuclei (Fig. 6d). However, H3K9, H4K16, and H4 exhibited different histone-lysine-acetylation patterns than H3K27 (Fig. 6d). Therefore, the FABP7-regulated nuclear environment appears to have a role in nonspecific histone lysine acetylation.

We then assessed whether FABP7 localization affected nuclear acetyl-CoA levels. Acetyl-CoA levels were increased in FABP7, FABP7-NLS, and FABP7-NES overexpressing whole cells (Fig. 6e). Notably, FABP7 and FABP7-NLS overexpression increased acetyl-CoA levels in purified functional nuclei (Fig. 6f; S5). Surprisingly, FABP7-NES overexpression did not alter acetyl-CoA levels (Fig. 6f). These results suggest that nuclear FABP7 levels are crucial for nuclear acetyl-CoA generation.

### FABP7 Interacts with and Regulates ATP-Citrate Lyase

**Fig. 7:**
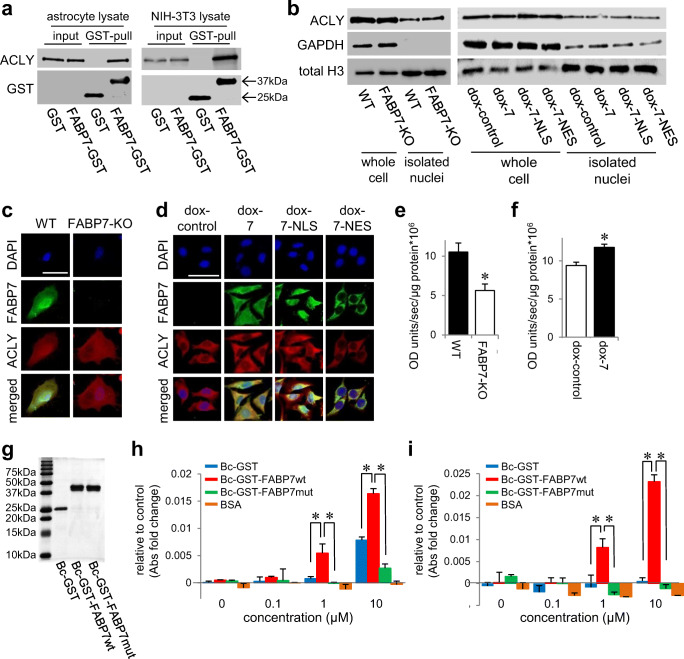
FABP7 interacts and regulates ATP-citrate lyase. **a** GST pull-down analysis and western blot to confirm the interaction of FABP7 and ACLY using FABP7-KO primary cultured astrocyte and NIH-3T3 cell lysate. **b** Western blot for ACLY expression in whole cell or isolated nuclei of WT and FABP7-KO primary cultured astrocytes or of NIH-3T3 cells with doxycycline-induced control, FABP7, FABP7-NLS, and FABP7-NES. **c**, **d** Immunofluorescence staining of FABP7 (green), ACLY (red), and DAPI (blue) in WT and FABP7-KO primary cultured astrocytes (**c**) or in NIH-3T3 cells with doxycycline-induced control, FABP7, FABP7-NLS, and FABP7-NES (**d**). Scale bar: 50 μm. **e**, **f** Measurement for ACLY activity in WT and FABP7-KO primary cultured astrocytes (**e**) or in NIH-3T3 cells with doxycycline-induced control and FABP7 (**f**). The levels were normalized by the protein concentration. **g** Western blot using GST antibody to confirm recombinant protein. **h**, **i** Measurement for ACLY activity with recombinant protein and BSA using FABP7-KO primary cultured astrocytes lysate (**h**) or NIH-3T3 cell lysate (**i**). Graph shows the difference compared to non-treated. Raw data is shown in Fig. S4e and S4f. Data shown are the means ± s.e.m. and representative of 3 independent experiments. *p < 0.05 versus WT or control. For Fig. 7**h** and **i**, analysis was performed in the group of between GST-treated and FABP7wt treated or between FABPwt-treated and FABP7mut-treated

To explore the molecular mechanism of FABP7 to modulate the acetyl-CoA levels, we performed immunoprecipitation and mass spectrometry analysis using HEK293T cells overexpressed with Flag-tagged human FABP7. We found 356 FABP7-interacting proteins (for the top 28, see Table 2). One of them was ATP-citrate lyase (ATP-citrate synthase, ACLY), an essential protein for nuclear acetyl-CoA generation. Pull-down assay confirmed the binding of FABP7 with ACLY in primary cultured astrocytes and NIH-3T3 cells (Fig. 7a). After confirming that FABP7 expression does not affect ACLY protein expression or cellular localization (Fig. 7b–d), we used the malate dehydrogenase coupled method to evaluate ACLY enzymatic activity in the presence or absence of FABP7 [42, 43]. ACLY activity in WT astrocytes and FABP7-overexpressed NIH-3T3 cells was significantly higher than that in FABP7-KO and control cells, respectively (Fig. 7e, f).

**Table 2:** Proteins interacted with FLAG-human FABP7wt from HEK293 cells

Gene name	Protein name	Protein score^1^	Spectral count^2^	Sequence^3^	Spectral count in mock^4^
	FLAG-humanFABP7	158,094	4827	31	6
PKP1	Plakophilin-1	828	31	7	–
PRDX1	Peroxiredoxin-1	545	29	7	
ALB	Serum albumin	372	20	5	
LGALS7	Galectin-7	423	11	4	–
EPPK1	Epiplakin	312	17	12	–
SERPINB3	Serpin B3	295	14	4	–
SERPINB4	Serpin B4	291	16	5	–
ACTN4	Alpha-actinin-4	287	13	5	–
HNRNPC	Heterogeneous nuclear ribonucleoproteins C1/C2	256	6	2	–
ATP5B	ATP synthase subunit beta, mitochondrial	255	22	5	1
TUBA1A	Tubulin alpha-1A chain	246	8	5	–
HIST1H2BB	Histone H2B type 1-B	219	7	3	–
HSPB1	Heat shock protein beta-1	188	9	6	–
SFN	14-3-3 protein sigma	166	10	4	–
PLP2	Proteolipid protein 2	157	7	1	–
HSP90AB1	Heat shock protein HSP 90-beta	156	8	4	
PARP1	Poly [ADP-ribose] polymerase 1	148	4	2	–
GSN	Gelsolin	144	8	5	–
HIST1H1D	Histone H1.3	138	13	5	–
RPS12	40S ribosomal protein S12	128	3	1	
TGM1	Protein-glutamine gamma-glutamyltransferase K	111	3	1	–
IGHG1	Immunoglobulin heavy constant gamma 1	108	9	2	
CAPG	Macrophage-capping protein	96	5	2	–
PSMA6	Proteasome subunit alpha type-6	94	4	2	
ACLY	ATP-citrate synthase	83	3	1	–
HIST1H2AB	Histone H2A type 1-B/E	79	5	2	
TYMP	Thymidine phosphorylase	75	4	2	–
DSG3	Desmoglein-3	67	2	2	–

Shown are representative proteins interacted with FABP7wt and without FABP7mut, with their MASCOT protein scores, spectral counts, and number of sequence. False discovery rate (FDR) calculated by MASCOT decoy search was 3.10%

^1^Protein score was determined by the MASCOT software

^2^Number of uniquely assigned spectral count for the respective proteins is shown

^3^Number of sequence match for the respective proteins is shown

^4^Number of spectral count for the respective proteins in the mock purification is shown

Next, we investigated whether FABP7 ligand binding is essential for interaction with ACLY. We prepared wild-type FABP7 and mutated FABP7 recombinant protein using the baculovirus expression system (Fig. 7g), and interestingly, the ligand-binding ability of mutated FABP7 was halved compared with wild-type FABP7 protein (Fig. S6a). Futhermore, LC-MS analysis revealed that mutated FABP7  could not bind ACLY (Table 3), leading us to the hypothesis that FABP7 ligand-binding capacity affects cellular ACLY activity.

**Table 3:** Proteins interacted with FLAG-human FABP7wt and without FLAG-human FABP7mut from HEK293 cells

Gene name	Protein name	Protein score^1^	Spectral count^2^	Sequence^3^	Spectral count in mock^4^	Spectral count in FLAG-human FABP7mut^5^
	FLAG-humanFABP7	158,094	4827	31	6	1519
PKP1	Plakophilin-1	828	31	7	–	3
LGALS7	Galectin-7	423	11	4	–	–
EPPK1	Epiplakin	312	17	12	–	–
SERPINB3	Serpin B3	295	14	4	–	–
SERPINB4	Serpin B4	291	16	5	–	–
ACTN4	Alpha-actinin-4	287	13	5	–	–
HNRNPC	Heterogeneous nuclear ribonucleoproteins C1/C2	256	6	2	–	–
ATP5B	ATP synthase subunit beta, mitochondrial	255	22	5	1	1
TUBA1A	Tubulin alpha-1A chain	246	8	5	–	1
HIST1H2BB	Histone H2B type 1-B	219	7	3	–	–
HSPB1	Heat shock protein beta-1	188	9	6	–	–
SFN	14-3-3 protein sigma	166	10	4	–	–
PLP2	Proteolipid protein 2	157	7	1	–	–
PARP1	Poly [ADP-ribose] polymerase 1	148	4	2	–	–
GSN	Gelsolin	144	8	5	–	–
TGM1	Protein-glutamine gamma-glutamyltransferase K	111	3	1	–	–
CAPG	Macrophage-capping protein	96	5	2	–	–
ACLY	ATP-citrate synthase	83	3	1	–	–
TYMP	Thymidine phosphorylase	75	4	2	–	–
DSG3	Desmoglein-3	67	2	2	–	–

Shown are representative proteins interacted with FABP7wt and without FABP7mut, with their MASCOT protein scores, spectral counts, and number of sequence. False discovery rate (FDR) calculated by MASCOT decoy search was 3.10%

^1^Protein score was determined by the MASCOT software

^2^Number of uniquely assigned spectral count for the respective proteins is shown

^3^Number of sequence match for the respective proteins is shown

^4^Number of spectral count for the respective proteins in the mock purification is shown

^5^Number of spectral count for the respective proteins in the FLAG-FABP7mut purification is shown

Moreover, mixing of wild-type FABP7 recombinant protein with FABP7-KO astrocyte lysates successfully increased ACLY activity, but mixing mutated FABP7 did not (Fig. 7h;S6b). We obtained similar results using NIH-3T3 cell lysates (Fig. 7i;S6c), suggesting that ligand-bound FABP7 interacts with ACLY and regulates its activity.

## Discussion

Acetyl-CoA is a fundamental component of de novo fatty acid synthesis and cholesterol. As an acetyl donor for histone lysine acetylation, acetyl-CoA is also an essential molecule for signaling and epigenetics [1]. In this study, we revealed that nuclear FABP7 is closely associated with nuclear acetyl-CoA levels, as well as H3K27ac levels on the promoters of several genes. These novel findings both improve understanding of a basic biological process, but also has important implications for tumor therapy, given that tumors require upregulated generation of acetyl-CoA due to their altered metabolism to obtain sufficient energy and components essential for rapid, uncontrolled proliferation.

Nuclear acetyl-CoA is generated via citrate-ACLY, acetate-ACSS2, and pyruvate-PDC pathway [5]. In this study, we verified that ACLY interacts with FABP7 (Tables 2 and 3, and Fig. 7a). Furthermore, ACLY co-localizes with FABP7 in both nucleus and cytoplasm (Fig. 7c, d). Thus, the FABP7-ACLY interaction may be crucial for maintaining nuclear acetyl-CoA levels and for epigenetic regulation of several genes. Interestingly, ACLY knockdown suppresses cell proliferation in several tumor cell lines, decreasing intracellular signaling (e.g., MAPK and Akt) in response to extracellular stimuli [42, 46, 47]. Likewise, FABP7 regulates the proliferation of several tumors [16, 48, 49]. In this study, we also found that FABP7-deficient astrocytes had decreased *Slc2a4* (insulin-responsive glucose transporter GLUT4) expression (Table 1); the gene is regulated by ACLY-related histone acetylation [5]. These phenotypic similarities strongly suggest that the FABP7-ACLY interaction modulates gene transcription via acetyl-CoA production. Consequently, the binding of these two proteins controls physiological and pathophysiological activity.

**Fig. 8:**
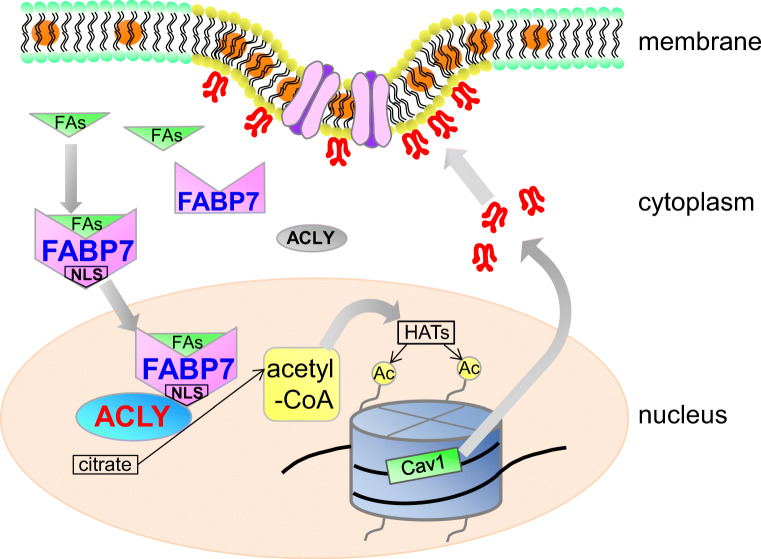
Schematic illustration depicting the putative functions of FABP7 in the astrocytes. FABP7 may bound with their ligands including fatty acids and recruit into nuclei forming the 3D-NLS-structure. In nucleus, FABP7 interacts with ACLY and upregulates the production of acetyl-CoA in nucleus, leading to histone acetylation of several gene promoter including caveolin-1

FABPs play an important role in transporting fatty acids to various subcellular compartments, including endoplasmic reticulum, mitochondria, and nucleus [50, 51], but the functional significance of FABP nuclear localization remains unknown. Previous research examining FABP7 three-dimensional protein structure found that the NLS is only observable in the helix-loop-helix region when protein is bound to activating ligands. Additionally, amino acids important for 3D-NLS are lysine at position 21, arginine at position 30, and glutamine at position 31 [50, 51]. Another study demonstrated that a mutation in the lipid-binding domain eliminates both FABP7 lipid-binding capacity and its nuclear localization, consequently decreasing tumor proliferation [16]. These data suggest that FABP7 ligand binding and its simultaneous translocation into the nucleus due to 3D-NLS are critical for nuclear FABP7 functions. Indeed, the mutation in FABP7 lipid binding domain did not enhance ACLY activity or acetyl-CoA levels (Fig. 7h, i). These data suggest that translocation of ligand-bound FABP7 into nuclei is important for nuclear acetyl-CoA metabolism and tumor proliferation possibly through epigenetic regulation of tumor proliferation related genes including caveolin-1 (Fig. 8).

FABP7 is highly expressed in patients with malignant glioma; expression levels are positively correlated with worsening pathological grade and poor prognosis [48, 52, 53]. The GEPIA from The Cancer Genome Atlas and GTEx consortiums also indicate significant ACLY upregulation in glioma (Fig. S7a, and S7b). These findings suggest that the FABP7 and ACLY interaction is a potential therapeutic target for malignant glioma. Recent research identified ACSS2 as a protein involved in glioma tumorigenesis via maintaining nuclear acetyl-CoA levels and activating lysosomal and autophagosomal gene expression in low-oxygen and low-glucose conditions [54, 55]. However, we did not observe any changes to ACSS2-related gene expression (data not shown). Glioma cells are highly likely to use different nuclear acetyl-CoA regulatory mechanisms depending on cellular nutritional environment (e.g., glucose and lipid content). Thus, clarifying the epigenetic involvement of ACLY-FABP7 and ACSS2 under different conditions is important for understanding glioma tumorigenesis. Research in this direction may provide clues for the development of new therapies targeting malignant glioma.

## Electronic Supplementary Material

ESM 1 (PDF 588 kb)

ESM 2 (DOCX 17 kb)

## Abbreviations

ACC: Acetyl-CoA carboxylase
ACLY: ATP-citrate lyase
ACSS2: Acyl-coenzyme A synthetase short-chain family member 2
ALA: α-Linolenic acid
APC: Allophycocyanin
DAPI: 4′,6-Diamidino-2-phenylindole, dihydrochloride
DHA: Docosahexaenoic acid
Egfr: Epidermal growth factor receptor
EPA: Eicosapentaenoic acid
FABP: Fatty acid binding protein
FASN: Fatty acid synthase
GAPDH: Glyceraldehyde 3-phosphate dehydrogenase
GFAP: Glial fibrillary acidic protein
GLAST: Glutamate aspartate transporter
GST: Glutathione S-transferase
Itgam: Integrin subunit alpha M (Cd11b)
KATs: Lysine acetyltransferases
LPL: Lipoprotein lipase
Mbp: Myelin basic protein
NeuN: Neuronal nuclei
PDC: Pyruvate dehydrogenase complex
PPAR: Peroxisome proliferator–activated receptor
PUFAs: Polyunsaturated fatty acids
SCPEP1: Serine carboxypeptidase 1
