# Supplementary material for: FABP7 Regulates Acetyl-CoA Metabolism Through the Interaction with ACLY in the Nucleus of Astrocytes
Source: Mol Neurobiol. 2020 Aug 19;57(12):4891–910. doi: 10.1007/s12035-020-02057-3 (PMC7541391; doi:10.1007/s12035-020-02057-3)
Supplement: Supplementary file 1 — (PDF 588 kb) [file 12035_2020_2057_MOESM1_ESM.pdf]

Supplemental Figure 1

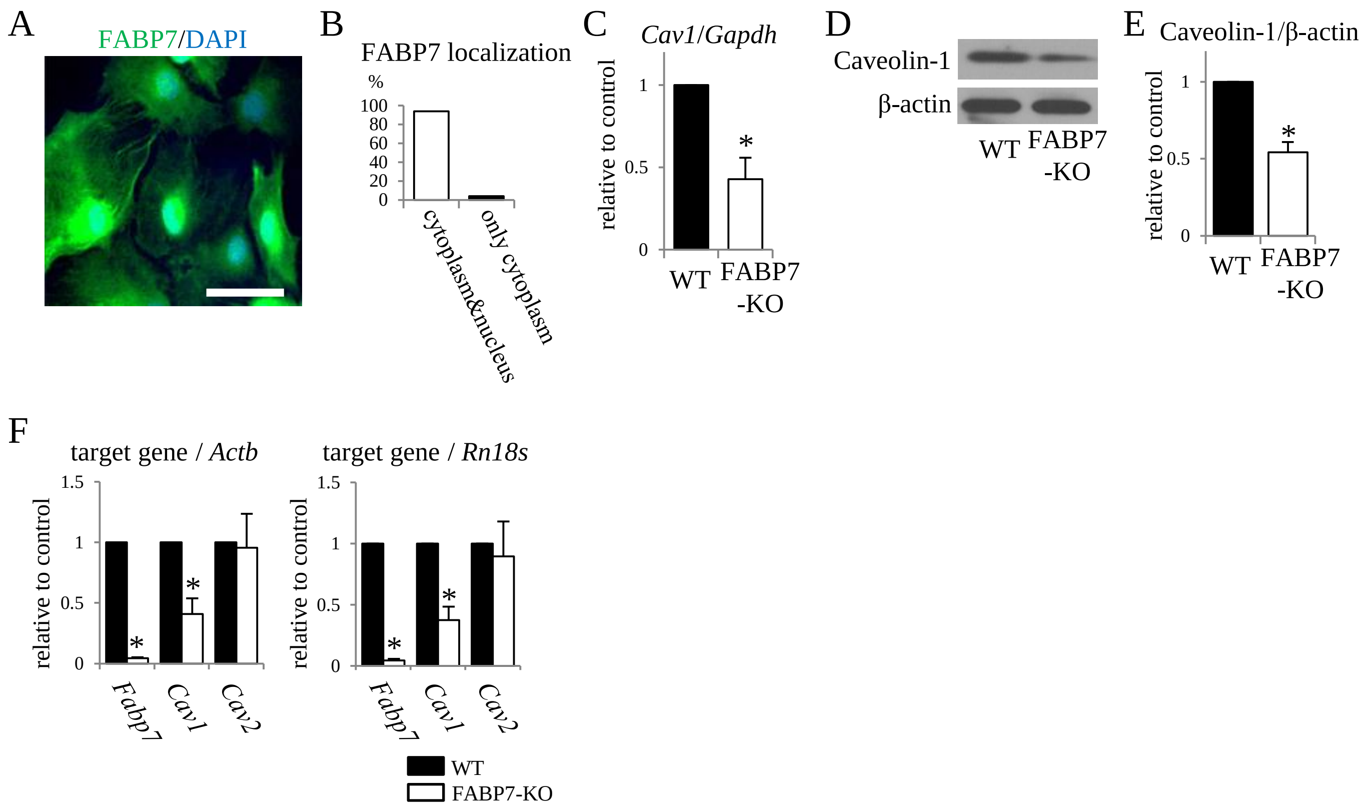

Figure S1. Related to Figure 1 (A) Immunofluorescence staining of FABP7 (green) and DAPI (blue) in primary cultured astrocytes. (B) Bar graph for the analysis of FABP7 localization in primary astrocytes. (C) qPCR analysis for *Cav1* expression in WT and FABP7-KO primary cultured astrocytes. (D, E) Western blot for caveolin-1 protein expression in WT and FABP7-KO primary cultured astrocytes. Bar graph (E) shows band density analyzed using NIH-Image J. (F) qPCR analysis for *Fabp7*, *Cav1* and *Cav2* expression in sorted astrocytes from WT and FABP7-KO mouse prefrontal cortex. *Actb* and *Rn18s* are used as endogenous genes. Data shown are the means ± s.e.m. and representative of 3 independent experiments. *p<0.05 versus WT.

Supplemental Figure 2

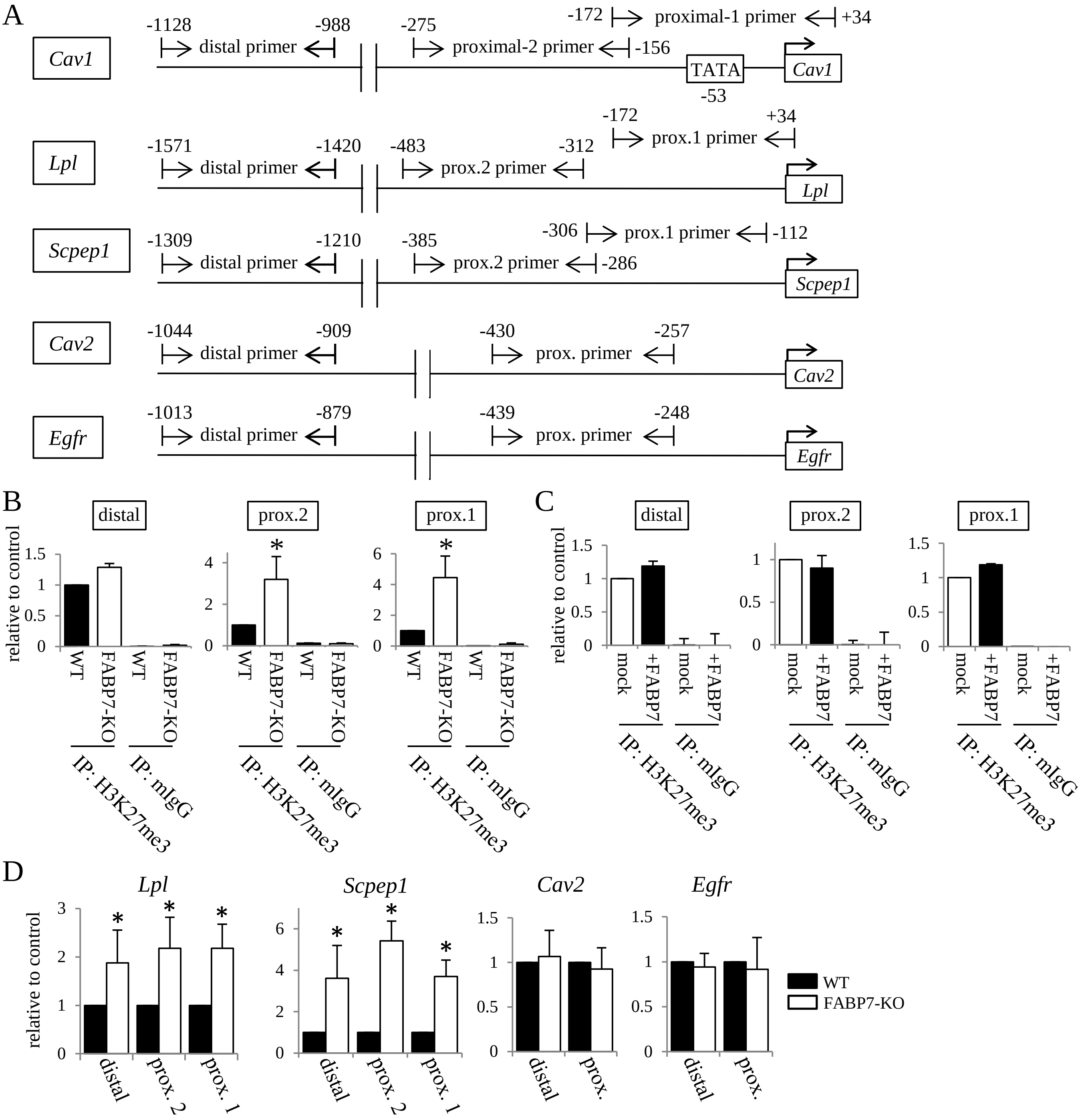

Figure S2. Related to Figure 3 (A) Schematic diagram of *Cav1*, *Lpl*, *Scpep1*, *Cav2* and *Egfr* promoter indicating the different primer sets used for ChIP analysis (B, C) ChIP assays and subsequent qPCR for H3K27me3 in primary cultured astrocytes (B) and NIH-3T3 cells (C). (D) ChIP assays and subsequent qPCR for H3K27me3 on each promoter region of WT or FABP7-KO primary cultured astrocytes. Data shown are the means ± s.e.m. and representative of 3 independent experiments. *p<0.05 versus WT or mock.

Supplemental Figure 3

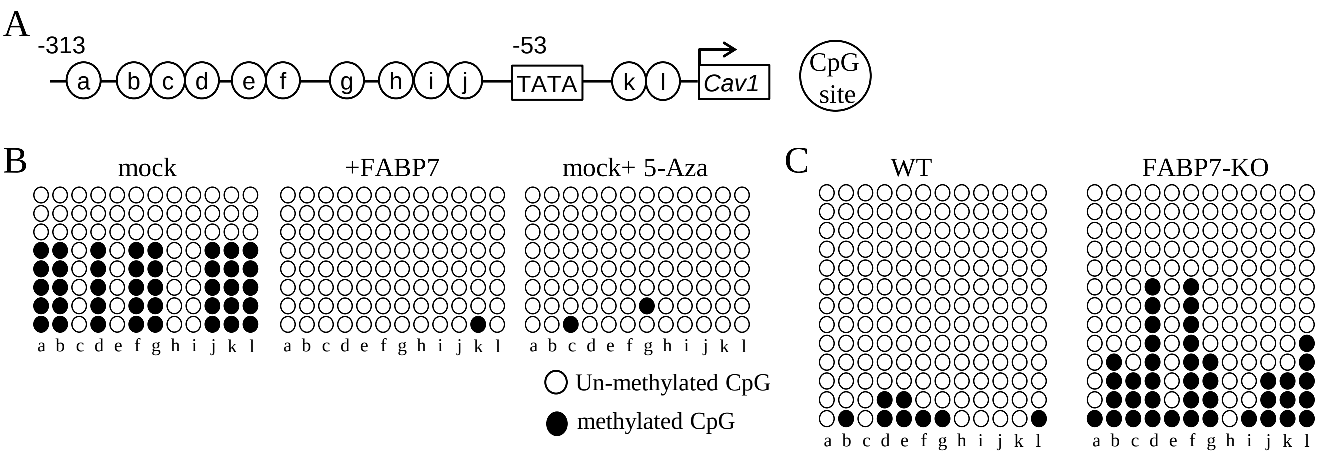

Figure S3. (A) Schematic representation of *Cav*1(-313/-1) promoter region with encircled CpG-rich sites. (B, C) Bisulfite sequence analysis in NIH3T3 cells with or without FABP7 expression (B) or in WT and FABP7-KO astrocytes, showing DNA methylation status. Mock cells treated with 5-Azacitidine was used a negative control. Data shown are representative of 3 independent experiments.

Supplemental Figure 4

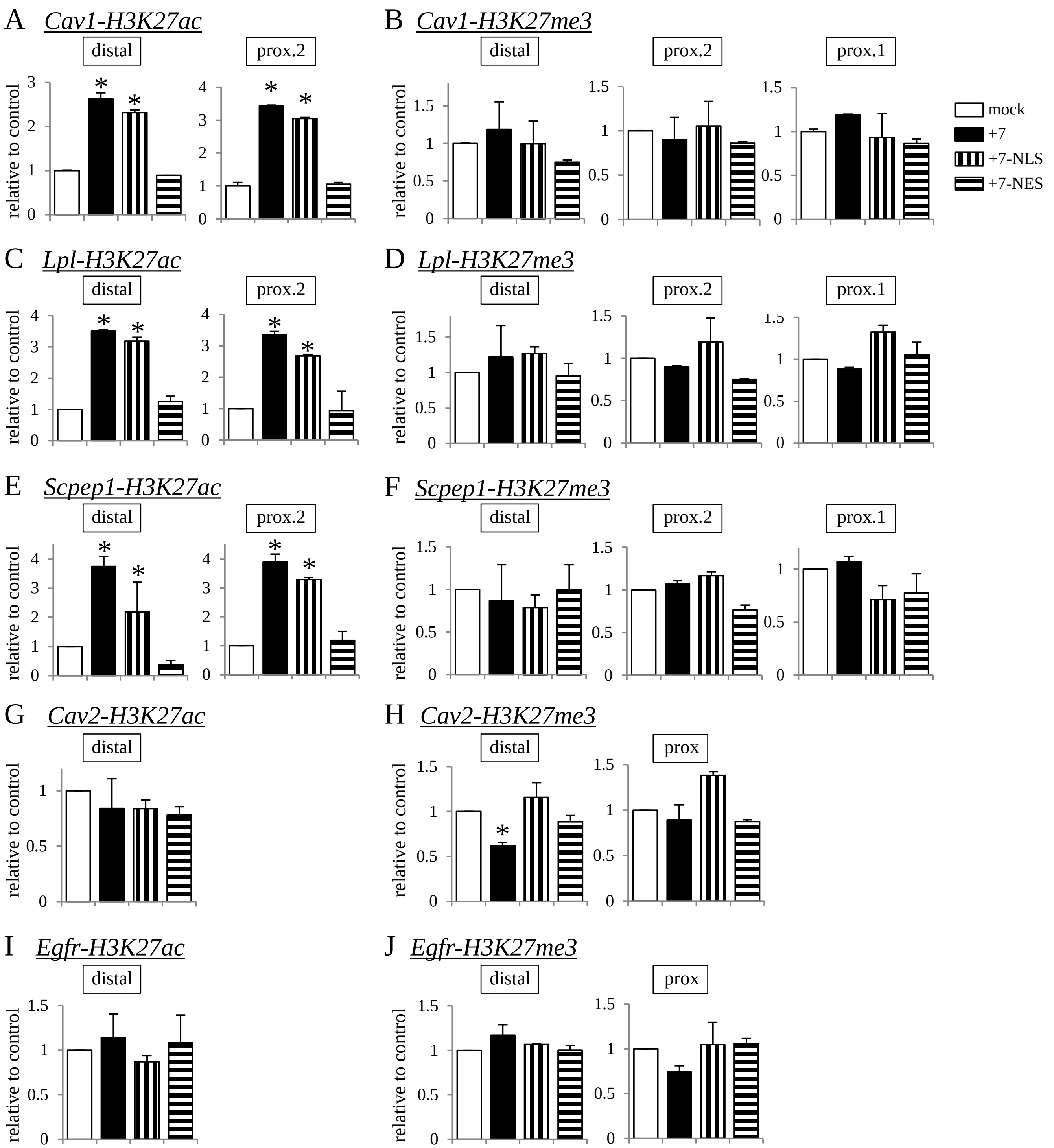

Figure S4. Related to Figure 4 (A-J) ChIP assays and subsequent qPCR for H3K27ac (A, C, E, G, I) and H3K27me3 (B, D, F, H, J) on each promoter region in NIH3T3 cells transfected with mock, ubiquitous FABP7, FABP7-NLS (N terminus), and FABP7-NES (N terminus) Data shown are the means ± s.e.m. and representative of 3 independent experiments. *p<0.05 versus mock.

Supplemental Figure 5

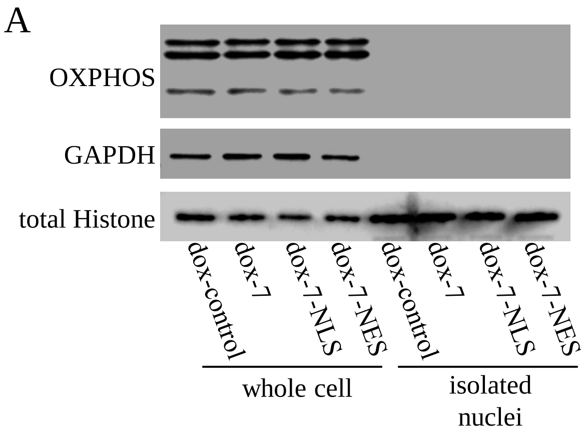

Figure S5. Related to Figure 6 (A) Western blot to confirm the purification of functional nuclei from NIH-3T3 cells with doxycycline-induced control, FABP7, FABP7-NLS, and FABP7-NES. OXPHOS, GAPDH and histone H3 are used as the marker of mitochondria, cytoplasm and nuclei, respectively.

Supplemental Figure 6

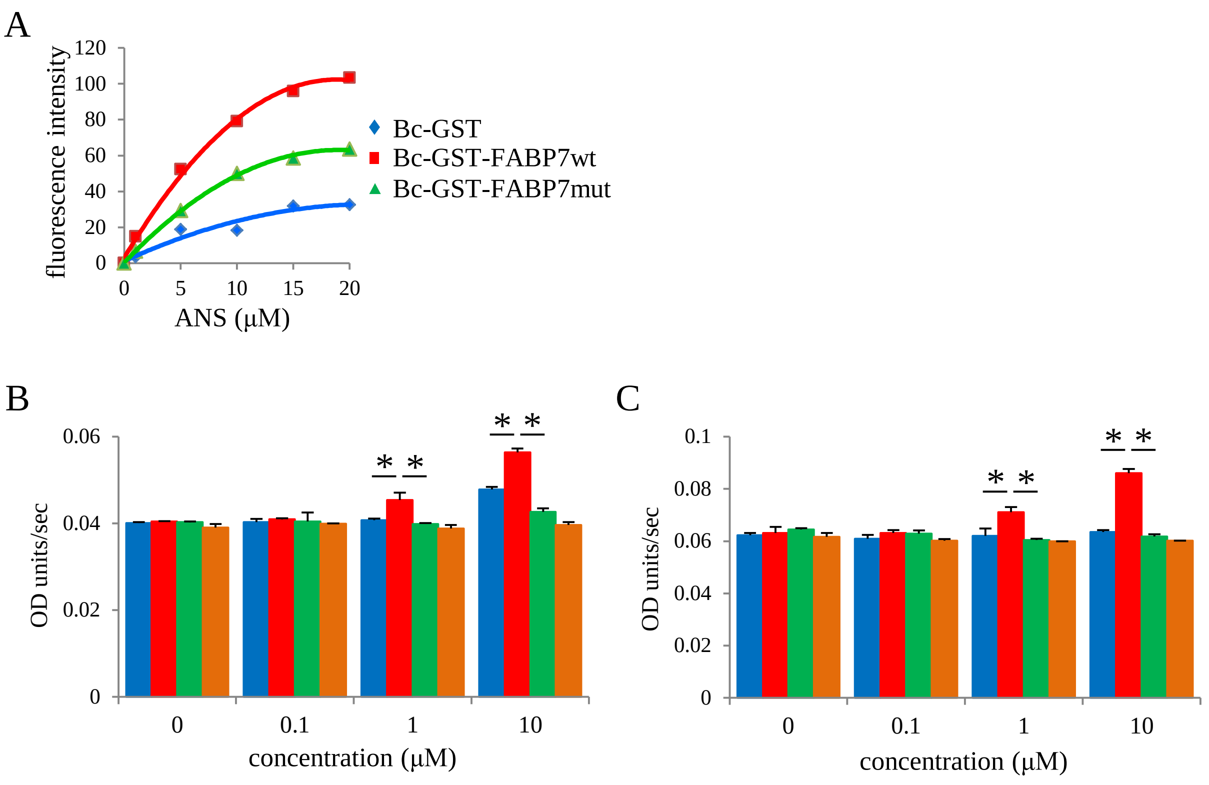

Figure S6. Related to Figure 7 (A) ANS binding assay to confirm the ligand-binding ability for constructed recombinant proteins. (B, C) Measurement for ACLY activity with recombinant protein and BSA using FABP7-KO primary cultured astrocytes (B) or NIH-3T3 cell lysate (C). Data shown are the means ± s.e.m. and representative of 3 independent experiments. *$p<0.05$

Supplemental Figure 7

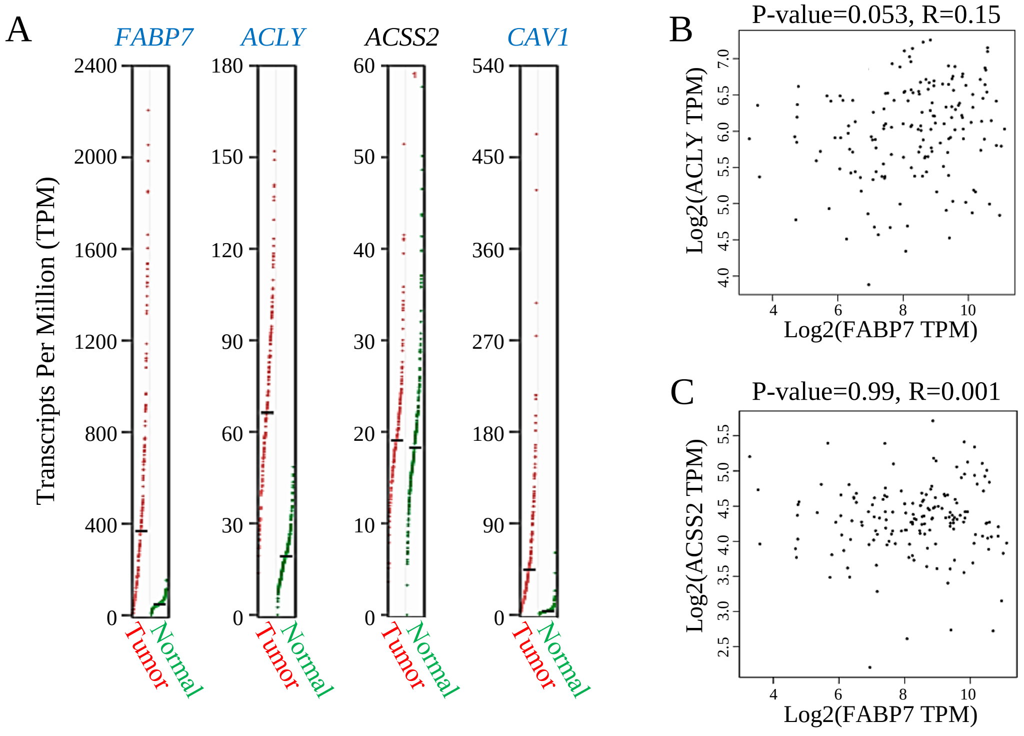

Figure S7. (A) gene expression analysis of human samples using a pubic databases (GEPIA). Expression in healthy tissues (green dots, data from GTEx xonsortium) or tumor tissues (red dots, data from TCGA consortium) was compared for GBM tumors. Each dot represents a patient/donor (n=163 GBM tumors;n=207 normal brain). Blue color of genes denotes significant upregulation in tumors (ANOVA; p<0.05).
