# Supplementary material for: FABP7 Regulates Acetyl-CoA Metabolism Through the Interaction with ACLY in the Nucleus of Astrocytes
Source: Mol Neurobiol. 2020 Aug 19;57(12):4891–910. doi: 10.1007/s12035-020-02057-3 (PMC7541391; doi:10.1007/s12035-020-02057-3)
Supplement: Supplementary file 2 — (DOCX 17 kb) [file 12035_2020_2057_MOESM2_ESM.docx]

**Supplemental Table 1. The list of oligonucleotides used in this study**

| purpose | sequence |
| --- | --- |
| Forward primer for mouse FABP7-NLS-N terminus | AGCAAAGCTTACCATGCCAAAGAAGAAGCGAAAGCTGGTAGATGCTTTCTGCGCAACCTGGAAGCTG |
| Reverse primer for mouse FABP7-NLS-N terminus | TGCTGAATTCCTATGCCTTTTCATAACAGCGAACAGC |
| Forward primer for mouse FABP7-NLS-C terminus | TATAGAATTCACCATGGTAGATGCTTTCTGC |
| Reverse primer for mouse FABP7-NLS-N terminus | TATACTCGAGCTACAGCTTTCGCTTCTTCTTTGGTGCCTTTTCATAACAGCGAACAGCAACG |
| Forward primer for mutation of Cav1 (-233, -229) | CCCGCCGGCACTCCTTTTTCTCTGCTGCCAGAACC |
| Reverse primer for mutation of Cav1 (-233, -229) | GGTTCTGGCAGCAGAGAAAAAGGAGTGCCGGCGGG |
| Forward primer for mutation of Cav1 (-116, -110) | CTCCCTCCCAGCCACTTTTTTCCGCCAGCGCCTTTCCCCC |
| Reverse primer for mutation of Cav1 (-116, -110) | GGGGGAAAGGCGCTGGCGGAAAAAAGTGGCTGGGAGGGAG |
| Forward primer for mutation of Cav1 (-108, -102) | CCAAGAGGCTCCCTCCCAGCCACTTTTTTCTTTTTTTGCCTTTCCCCCCTCTATACAATACAA |
| Reverse primer for mutation of Cav1 (-108, -102) | TTGTATTGTATAGAGGGGGGAAAGGCAAAAAAAGAAAAAAGTGGCTGGGAGGGAGCCTCTTGG |
| Forward primer for bisulfite sequence analysis | TTTTTTGTAGGTTTATAGTTGGGAAAA |
| Reverse primer for bisulfite sequence analysis | ATAACAAAAACAAACACTTTAAAACCCT |
| Forward primer for CHIP assay of Cav1 distal | TTTCATCCTTGAGGTGGTG |
| Reverse primer for CHIP assay of Cav1 distal | CAGTTTCAAAAGCTGGCC |
| Forward primer for CHIP assay of Cav1 proximal2 | TGAAGGCTTTCTCACAGGC |
| Reverse primer for CHIP assay of Cav1 proximal2 | TGTTCTGCTCTCAGTTGGC |
| Forward primer for CHIP assay of Cav1 proximal1 | CAACTGAGAGCAGAACAAACC |
| Reverse primer for CHIP assay of Cav1 proximal1 | CTACCTCGGAGTCTACGTATTTG |
| Forward primer for CHIP assay of Lpl distal | TCAGTGGGGTTTCGCTCTTG |
| Reverse primer for CHIP assay of Lpl distal | GCCAAACATGCTGTTGTGTAG |
| Forward primer for CHIP assay of Lpl proximal2 | GACTACTCAACATAAATCAGTG |
| Reverse primer for CHIP assay of Lpl proximal2 | TTGAGCAAACGGTAACGAGGC |
| Forward primer for CHIP assay of Lpl proximal1 | AGCTGTGCAGTGGAAACAGT |
| Reverse primer for CHIP assay of Lpl proximal1 | GCTTTGCTGCTGGAACTACG |
| Forward primer for CHIP assay of Scpep1 distal | TTCAAATTCCTCTCCACAAGCC |
| Reverse primer for CHIP assay of Scpep1 distal | TCTCTCTTGATAGCATGGTCAAC |
| Forward primer for CHIP assay of Scpep1 proximal2 | TCCTTCTGCATAAACCCACAC |
| Reverse primer for CHIP assay of Scpep1 proximal2 | GCGTGTTTGTCTTCTCCGAC |
| Forward primer for CHIP assay of Scpep1 proximal1 | TGTCGGAGAAGACAAACACGG |
| Reverse primer for CHIP assay of Scpep1 proximal1 | CTCCTAAGGATTCCGCGCTC |
| Forward primer for CHIP assay of Cav2 distal | ATGCATGTAGACTTCCCTAATGC |
| Reverse primer for CHIP assay of Cav2 distal | CTCGATGAGTCATCTCCTGGC |
| Forward primer for CHIP assay of Cav2 proximal | TTTGGCAAACAACTGGAGAG |
| Reverse primer for CHIP assay of Cav2 proximal | GGCTCGGTCGTTCGGTCCCTAG |
| Forward primer for CHIP assay of Egfr distal | GAGCCTTGTCTAGTGGTGGC |
| Reverse primer for CHIP assay of Egfr distal | CAGACGTCTAGTTAGCTCGGG |
| Forward primer for CHIP assay of Egfr proximal | CCGCCTCCCAGACAGACGACAGG |
| Reverse primer for CHIP assay of Egfr proximal | CCTGAGGGTCGCATCTCTCTGACC |
